# Exploring wild rices for photosynthetic efficiency improvement in rice

**DOI:** 10.1007/s12298-026-01761-z

**Published:** 2026-06-13

**Authors:** Kanchan Jumrani, Dipti Ranjan Pani, Mridul Chakraborti, Mirza Jaynul Baig, Viswanathan Chinnusamy, Palanisamy Veeraya, Ritu Tiwari, Dhammaprakash P. Wankhede, Rajkumar Subramani, Kanakasabapathi Pradheep, Parimalan Rangan

**Affiliations:** 1 ICAR-National Bureau of Plant Genetic Resources, PUSA Campus, New Delhi, 110012 India; 2 ICAR-National Bureau of Plant Genetic Resources, Base Centre, Cuttack, 753006 India; 3 ICAR-National Rice Research Institute, Cuttack, 753006 India; 4 ICAR-Indian Agricultural Research Institute, PUSA Campus, New Delhi, 110012 India; 5 ICAR-National Bureau of Plant Genetic Resources, Regional Station, Thrissur, 680656 India; 6 Queensland Alliance for Agriculture and Food Innovation, University of Queensland, Brisbane, QLD 4072 Australia

**Keywords:** Chlorophyll fluorescence, Domestication syndrome, Gas exchange, Photosynthesis, *Oryza*, Stomatal conductance

## Abstract

Photosynthesis is a key determinant for plant productivity, understanding its genetic basis will aid its improvement. The photosynthesis trait is primarily measured in terms of carbon and light utilization, using gas exchange and chlorophyll fluorescence methods. We report in this study, the extent of variability for photosynthetic trait in wild rice relatives, comprising a total of 26 taxa (22 wild species), using an infrared gas analyzer. The scale of measures used were, PN (net photosynthetic rate), E (transpiration), gs (stomatal conductance), Ci (intercellular CO_2_), Ci/Ca (ratio of intercellular to ambient CO_2_), WUE (water use efficiency), CE (carboxylation efficiency), Fv′/Fm′ (ratio of variable to maximum fluorescence, photosynthesis efficiency), qP (photochemical quenching), qN (non-photochemical quenching), ΦPSII (quantum yield of photosystemII), and ETR (electron transport rate). These were subjected to variability (ANOVA and PCA) and association (correlation matrices and path analysis) studies. The maximum PN was observed in *Oryza* *australiensis*, followed by *Oryza* *officinalis* and *Oryza* *barthii*. Maximum qP was observed in *Oryza latifolia* which was at par with *O*. *australiensis*, and *Oryza* *alta*. Highest CE was observed in *O*. *australiensis*. The CE values for *O*. *barthii*, *Oryza grandiglumis* and *O. latifolia* were at par with* O*. *australiensis*. Transcriptomic analysis for flag leaf between APO, the high efficient photosynthetic genotype with indica rice cultivar BAM4234 revealed key candidate genes underlying photosynthesis traits. These findings underscore the value of wild species and their alleles in expanding the photosynthetic capacity of rice potentially using classical breeding approaches.

**Supplementary Information:**

The online version contains supplementary material available at 10.1007/s12298-026-01761-z.

## Introduction

Rice (*Oryza sativa* L.) is one of the most important staple crops, and feeds nearly half of the world’s population (Li et al. 2018; Mohidem et al. 2022; Yiwei et al. 2024; Zhang et al. 2025; Haoran et al. 2025). China and India had produced 144.62 and 137.83 million tons of rice that account for 28% and 26%, respectively of the global rice production (FAO 2024; Shahbandeh 2024). In the context of global warming and increasing food demand worldwide (1.7% annually), research on sustainable agriculture and enhanced productivity for staple crops has become more crucial than ever. Several studies project that global crop production must be scaled up by 2050 to meet the growing food demand driven by population growth, dietary shifts toward more resource-intensive foods, and rising biofuel needs (Kondamudi et al. 2016; Hunter et al. 2017; Walkowiak et al. 2020; Van Dijk et al. 2021). Genetic improvement through harvesting ‘harvest index’ is getting saturated, and the next in line scope for improvement is through enhancing the light harvest and carbon fixation, the photosynthesis (South et al. 2018; Furbank et al. 2020; Mathan et al. 2021a). Estimates suggest that improving photosynthetic efficiency could boost rice yields by 10–30% (Xiong 2024).

Photosynthesis is an important biological process that sustains all life forms on this planet Earth (Uflewski et al. 2024). The complex photochemical and biochemical processes of photosynthesis majorly occur in chloroplast organelles and are generally split into two stages: the light reaction and the dark reaction (Shevela et al. 2019). The light reaction, also known as the “photosynthetic electron transport reaction”, transforms light energy into chemical energy at the thylakoid membrane (Sujatha 2015). The dark reactions of photosynthesis, also known as the Calvin cycle, is a series of biochemical processes that transforms the chemical energy obtained from light reaction into organic compounds through carbon assimilation in the stroma of the chloroplast organelles in plant cells (Flugge et al. 2016). In cereal crops, the flag leaf (source) plays a crucial role in carbon translocation to the developing inflorescence (sink) and is widely recognized as a major source of photoassimilate for grain development (Tanaka et al. 2022). The genetic variation for photosynthetic traits in both cultivated and wild species includes potential to improve crop photosynthesis and ultimately productivity through introgression of alleles (from wild) associated with enhanced photosynthetic efficiency (Burgess et al. 2023; Garcia et al. 2023).

Crop wild relatives (CWRs) harbor more allelic diversity than cultivars and are known for its genetic treasure trove with valuable genes and alleles associated with biotic and abiotic tolerance traits, nutritional quality, and higher rate of dry matter accumulation (Rangan et al. 2023; Padmavathi et al. 2024). A pan-tropical genus, *Oryza*, includes two cultivated species *O. sativa* (Asian rice, with subsp./var., *indica*, *japonica*, and *javanica*) and *O*. *glaberrima* (African rice); and 24 wild species (Brar and Khush 2018; Stein et al. 2018; Fornasiero et al. 2024). Higher photosynthetic performance in wild rice species as compared to cultivated ones were well documented (Kiran et al. 2013; Giuliani et al. 2013; Acevedo-Siaca et al. 2020; Mathan et al. 2021b; Phillips et al. 2022). Though wild species often exhibit higher photosynthetic rates, they exhibit a lesser harvest index than the domesticated ones (Acevedo-Siaca et al. 2021). We report here the variability for photosynthesis and chlorophyll fluorescence measures studied in 22 wild relatives and 2 cultivated rice species (26 taxa).

## Materials and methods

### Plant material

The observations were recorded from the wild *Oryza* plants (26 taxa) (Supplementary Table S1) being maintained at the wild rice garden jointly by the ICAR-CRRI and ICAR-NBPGR Base Centre, Cuttack, Odisha (20.4625° N, 85.8830° E). Plants were grown under natural field conditions with raised (from ground level) concrete rings (120 cm dia. and 45 cm ht.—of which ca. 5 cm is below soil so above ground is ca. 40 cm, with ring thickness being 4 cm) with an average air temperature and relative humidity recorded as 30 °C and 70–80%, respectively. The measurements were taken from the middle portion (with respect to length) of the fully expanded flag leaf at anthesis stage. We chose to measure at this stage since the flag leaves were known to make maximal contribution for yield than the other lower leaves. The total number of plants maintained per taxa were three, and each plant was treated as a biological replicate. Each biological replicate value is an average of the five measurements from a flag leaf for each accession.

### Data collection

#### Gas exchange measurements

Leaf photosynthetic parameters were measured between 10:00 and 12:00 h on fully matured flag leaves using a portable photosynthesis measuring system (LI-6400 XT, LI-COR Inc., Lincoln, NE, USA). The flag leaf of the rice plants was fitted into 2 cm^2^ leaf chamber and the measurements were made at photosynthetically active radiation (PAR) of 1200 µmol m^−2^ s^−1^ at an ambient CO_2_ concentration (410–430 μmol mol^−1^ air CO_2_) with a constant airflow of 300 µmol s^−1^ and at a RH of 70–80%. During the measurement, leaf temperature was maintained at 25 °C. Net photosynthetic rate (PN), stomatal conductance (gs), transpiration rate (E), and intercellular CO_2_ (Ci) were measured. Water use efficiency (WUE) was calculated from inbuilt instantaneous values of PN and E. Carboxylation efficiency (CE) was obtained from PN and Ci. The Ci/Ca represents the ratio of intercellular CO_2_ to ambient CO_2_.

#### Chlorophyll fluorescence measurements

Chlorophyll fluorescence parameters were recorded in light adapted leaves, for which gas exchange was measured, using LI-6400 XT portable photosynthesis system combined with a 6400-40 leaf chamber fluorometer, which allows taking simultaneous measurement of gas exchange and chlorophyll fluorescence. Electron transport rate (ETR), Fv′/Fm′, photochemical quenching (qP), non-photochemical quenching (qN) and ΦPSII were measured.

### Transcriptome analysis

Using our previously reported transcriptome dataset generated for flag leaf tissue for three genotypes viz., APO (EC734333), BAM4234 (EC497171), and CROSSA (IC575838); RNA-seq analysis was performed between the genotypes and identified the set of significantly differentially regulated genes between the photosynthetically efficient genotype (APO) in comparison with the other genotypes. Readers may refer to Rangan et al. (2025) for detailed methodology on sample collection and transcriptome analysis. The data is accessible on the public domain through the project E-MTAB-8361.

### Statistical analysis

All the statistical analysis were performed using various packages of “R Studio” (v4.2). Analysis of variance (Supplementary Table S2 and S3) was carried out for all the data sets using “agricolae” package (de Mendiburu 2021) with *p* ≤ 0.05 as cut-off value for significance. Principal component analysis (PCA) analysis for the measured variables across 26 taxa was assembled using the “Factoextra”, “dplyr”, and “plotly” packages of R (Blighe 2021). For dendrogram analysis, clustering method was used based on the dissimilarity between the *Oryza* species using “cluster”, “pvclust” package (Maechler et al. 2023). Clustering was conducted using the Ward’s minimum variance method (ward D^2^) with Euclidean distance as the dissimilarity metric. To assess the robustness of the clusters, bootstrap resampling with 1000 iterations was performed. Approximately unbiased (AU) *p* values and bootstrap probability (BP) values were used to evaluate cluster support. Genotypic correlation and path analysis were performed using “Agri Analyze” online tool (Popat et al. 2024). The mean for each trait between the studied taxa was compared based on least significant differences (LSD) value using duncan multiple range test (DMRT) statistic. Additionally, multivariate analysis of variance (MANOVA; Supplementary Table S4) was carried out using ‘iris’, ‘car’ and ‘psych’ package in “R”. Permutational multivariate analysis (PERMANOVA; Supplementary Table S5) was also carried using the ‘vegan’ package with 999 permutations and Euclidean distances were chosen (Jari-Oksanen et al. 2019).

## Results

### Photosynthesis and associated parameters

Photosynthesis is a physiological process by which plants convert light energy, water, and CO_2_ into glucose and oxygen. CO_2_ is a key ingredient in this process and is a rate-limiting factor. Among the measures studied, net photosynthetic rate (PN) is the determining factor that reflects overall photosynthetic performance of the plant.

Significant variability was observed for PN among *Oryza* species studied. The PN values varied from 5.2 to 26.0 µmol CO_2_ m^−2^ s^−1^ with a mean value 15.0 (Table 1). Only one species, *O*. *australiensis*, had recorded PN value > 25 and *O*. *officinalis* (24.7) is on par with it.

**Table 1:** Photosynthesis rate (PN), stomatal conductance (gs), intercellular CO_2_ (Ci) and rate of transpiration (E) as measured in the flag leaf of the cultivated and wild rice species

S. No.	*Oryza* taxa	Photosynthesis rate (PN) (µmol CO_2_ m^−2^ s^−1^)	Stomatal Conductance (gs) (mol H_2_O m^−2^ s^−1^)	Intercellular CO_2_ (Ci) (µmol CO_2_ mol^−1^ air)	Transpiration rate (E) (mmol H_2_O m^−2^ s^−1^)
1.	*O. alta*	16.8 ± 0.9^e^	0.200 ± 0.019^ef^	254.9 ± 5.8^ghij^	5.3 ± 0.4^cd^
2.	*O. australiensis*	26.0 ± 0.1^a^	0.338 ± 0.002^b^	263.3 ± 0.3^efghi^	6.6 ± 0.1^b^
3.	*O. barthii*	23.1 ± 0.3^bc^	0.266 ± 0.009^cd^	246.8 ± 3.6^ij^	4.9 ± 0.3^cde^
4.	*O. brachyantha*	5.2 ± 0.0^j^	0.103 ± 0.002^ij^	312.9 ± 1.6^ab^	3.2 ± 0.1^hijk^
5.	*O. coarctata*	9.3 ± 0.3^i^	0.111 ± 0.005^hij^	284.6 ± 4.3^de^	1.9 ± 0.2^jkl^
6.	*O. eichingeri*	10.6 ± 0.4^hi^	0.116 ± 0.008^ghij^	254.7 ± 2.5^ghij^	3.1 ± 0.1^ijkl^
7.	*O. glaberrima*	10.7 ± 1.0^hi^	0.168 ± 0.020^fghi^	285.1 ± 4.6^de^	4.8 ± 0.6^cdef^
8.	*O. glumaepatula*	8.7 ± 0.6^i^	0.068 ± 0.006^j^	188.7 ± 4.1^l^	2.2 ± 0.2^kl^
9.	*O. grandiglumis*	22.9 ± 0.2^bc^	0.286 ± 0.008^bcd^	258.1 ± 2.3^fghij^	5.6 ± 0.3^bc^
10.	*O. granulata*	10.7 ± 0.3^hi^	0.135 ± 0.015^ghi^	260.5 ± 11.0^fghi^	3.2 ± 0.3^ijk^
11.	*O. latifolia*	21.0 ± 0.7^cd^	0.230 ± 0.016^de^	237.2 ± 4.7^j^	6.6 ± 0.4^b^
12.	*O. longiglumis*	22.6 ± 0.9^bcd^	0.292 ± 0.039^bc^	258.3 ± 9.2^fghij^	5.7 ± 0.7^bc^
13.	*O. longistaminata*	20.5 ± 0.7^d^	0.281 ± 0.016^bcd^	264.5 ± 9.5^efghi^	7.7 ± 0.4^a^
14.	*O. meridionalis*	10.5 ± 0.4^hi^	0.112 ± 0.005^hij^	243.5 ± 1.4^ij^	3.5 ± 0.2^ghij^
15.	*O. meyeriana*	10.2 ± 1.4^hi^	0.157 ± 0.010^fghi^	306.6 ± 8.1^abc^	3.0 ± 0.2^ijkl^
16.	*O. minuta*	9.4 ± 0.0^i^	0.132 ± 0.000^ghi^	274.9 ± 4.8^defgh^	3.2 ± 0.0^l^
17.	*O. nivara*	22.7 ± 1.1^bcd^	0.333 ± 0.040^b^	290.5 ± 7.1^cd^	5.1 ± 0.5^cd^
18.	*O. officinalis*	24.7 ± 0.9^ab^	0.507 ± 0.070^a^	325.9 ± 14.2^a^	7.9 ± 0.8^a^
19.	*O. punctata*	12.6 ± 0.4^gh^	0.161 ± 0.008^fghi^	276.0 ± 3.4^defg^	2.6 ± 0.0^jkl^
20.	*O. rhizomatis*	15.2 ± 0.3^ef^	0.175 ± 0.011^efgh^	251.7 ± 6.1^hij^	3.7 ± 0.2^fghi^
21.	*O. ridleyi*	10.0 ± 0.3^i^	0.144 ± 0.008^fghi^	281.1 ± 2.7^def^	4.2 ± 0.3^defgh^
22.	*O. rufipogon*	15.8 ± 1.4^ef^	0.144 ± 0.017^fghi^	211.9 ± 14.9^k^	4.3 ± 0.5^defgh^
23.	*O. spontanea*	10.8 ± 1.1^hi^	0.139 ± 0.009^fghi^	266.6 ± 9.5^efghi^	4.3 ± 0.1^defgh^
24.	*O. sativa* ssp*. japonica*	15.2 ± 0.8^ef^	0.135 ± 0.001^ghi^	211.4 ± 10.7^k^	3.1 ± 0.1^ijkl^
25.	*O. sativa* var*. javanica*	11.1 ± 0.5^hi^	0.169 ± 0.006^fghi^	272.3 ± 11.5^defgh^	4.4 ± 0.1^defg^
26.	*O. sativa* ssp*. indica*	13.9 ± 1.3^fg^	0.179 ± 0.004^efg^	293.5 ± 3.2^bcd^	3.9 ± 0.4^efghi^

Values represent mean ± SE, n = 3 data from different plants; each data point is an average of 5 values. Mean values ± SE between rows within a column were compared for significance and different letters indicate they are statistically significant at *p* ≤ 0.05

Photosynthesis and stomatal conductance (gs, the degree of stomatal opening) are closely linked and influence each other positively in a complex way. Significant differences were observed for gs between the *Oryza* species studied (Table 1). *O. officinalis* exhibited the maximum gs, 0.507 mol H_2_O m^−2^ s^−1^ followed by *O*. *australiensis* (0.338) and *O*. *nivara* (0.333).

The “intercellular CO_2_” refers to the CO_2_ concentration in the intercellular spaces between mesophyll cells of a leaf. Stomatal conductance is correlative with the Ci values. The Ci values (Table 1) ranged between 188.7 (*O*. *glumaepatula*) and 325.9 µmol CO_2_ mol air^−1^ (*O*. *officinalis*).

Transpiration in leaf can help to dissipate heat and hence make rate of photosynthesis optimal. Transpiration (E) values exhibited significant variation among the *Oryza* species studied (Table 1). The E values varied from 1.9 (*O. coarctata*) to 7.9 mmol H_2_O m^−2^ s^−1^ (*O*. *officinalis*) with a mean value 4.3.

The WUE is defined as the ratio of carbon fixed (via photosynthesis) to the water lost through transpiration. Significant variation for WUE was observed among *Oryza* species studied, and values were between 1.629 (*O. brachyantha*) and 4.960 mmol mol^−1^ (*O. sativa* subsp. *japonica*) (Table 2).

**Table 2:** Ci/Ca, water use efficiency (WUE), carboxylation efficiency (CE) as measured in the flag leaf of the cultivated and wild rice species

S. No.	*Oryza* taxa studied	Ci/Ca	Water use efficiency (PN/E)	Carboxylation efficiency (PN/Ci)
1.	*O. alta*	0.626 ± 0.015^fg^	3.160 ± 0.087^efgh^	0.066 ± 0.002^fg^
2.	*O. australiensis*	0.657 ± 0.001^def^	3.968 ± 0.085^bcde^	0.099 ± 0.000^a^
3.	*O. barthii*	0.618 ± 0.009^fg^	4.720 ± 0.242^abc^	0.094 ± 0.001^ab^
4.	*O. brachyantha*	0.756 ± 0.004^ab^	1.629 ± 0.045^i^	0.017 ± 0.000^f^
5.	*O. coarctata*	0.663 ± 0.009^def^	3.676 ± 0.279^cdef^	0.033 ± 0.001^ef^
6.	*O. eichingeri*	0.703 ± 0.016^bcde^	3.472 ± 0.223^defg^	0.042 ± 0.001^cdef^
7.	*O. glaberrima*	0.703 ± 0.012^bcde^	2.266 ± 0.153^hi^	0.037 ± 0.003^ef^
8.	*O. glumaepatula*	0.457 ± 0.010^j^	3.894 ± 0.052^bcde^	0.046 ± 0.003^hi^
9.	*O. grandiglumis*	0.645 ± 0.006^efg^	4.082 ± 0.212^bcde^	0.089 ± 0.001^abc^
10.	*O. granulate*	0.645 ± 0.028^efg^	3.453 ± 0.281^defgh^	0.041 ± 0.001^def^
11.	*O. latifolia*	0.590 ± 0.013^gh^	3.189 ± 0.082^efgh^	0.088 ± 0.001^abc^
12.	*O. longiglumis*	0.648 ± 0.025^ef^	4.060 ± 0.297^bcde^	0.087 ± 0.001^bcd^
13.	*O. longistaminata*	0.661 ± 0.025^def^	2.689 ± 0.245^fghi^	0.078 ± 0.005^cde^
14.	*O. meridionalis*	0.591 ± 0.004^gh^	3.019 ± 0.044^efgh^	0.043 ± 0.001^hij^
15.	*O. meyeriana*	0.726 ± 0.018^abc^	3.354 ± 0.267^defgh^	0.034 ± 0.005^ef^
16.	*O. minuta*	0.691 ± 0.024^cde^	2.973 ± 0.000^a^	0.034 ± 0.001^ef^
17.	*O. nivara*	0.703 ± 0.018^bcde^	4.518 ± 0.183^abcd^	0.078 ± 0.002^cde^
18.	*O. officinalis*	0.774 ± 0.032^a^	3.187 ± 0.400^efgh^	0.076 ± 0.006^def^
19.	*O. punctata*	0.658 ± 0.007^def^	4.902 ± 0.113^ab^	0.046 ± 0.002^hi^
20.	*O. rhizomatis*	0.621 ± 0.015^fg^	4.100 ± 0.170^bcde^	0.060 ± 0.001^g^
21.	*O. ridleyi*	0.684 ± 0.007^cde^	2.373 ± 0.079^ghi^	0.036 ± 0.001^ef^
22.	*O. rufipogon*	0.521 ± 0.036^i^	3.697 ± 0.279^cdef^	0.075 ± 0.010^def^
23.	*O. spontanea*	0.655 ± 0.022^def^	2.508 ± 0.205^fghi^	0.041 ± 0.006^def^
24.	*O. sativa* ssp*. japonica*	0.546 ± 0.013^hi^	4.960 ± 0.270^ab^	0.073 ± 0.007^ef^
25.	*O. sativa* var*. javanica*	0.707 ± 0.013^bcd^	2.557 ± 0.141^fghi^	0.041 ± 0.003^def^
26.	*O. sativa* ssp*. indica*	0.527 ± 0.011^i^	3.633 ± 0.295^cdef^	0.047 ± 0.004^h^

Values represent mean ± SE, n = 3 data from different plants; each data point is an average of 5 values. Mean values ± SE between rows within a column were compared for significance and different letters indicate they are statistically significant at *p* ≤ 0.05

Mathematically, carboxylation efficiency is the PN/Ci ratio, and it indicates how effectively RuBisCO is fixing the available CO_2_. Among all the *Oryza* species studied, maximum CE was observed in *O*. *australiensis* (0.099 μmol CO_2_ m^−2^ s^−1^/μmol CO_2_ mol^−1^ air). The CE values for *O*. *barthii* (0.094) *O. grandiglumis* (0.089) and *O. latifolia* (0.089) were at par with the maximum CE (Table 2).

Ratio of intercellular CO_2_ (Ci) to ambient CO_2_ (Ca) is represented as Ci/Ca. Maximum and minimum Ci/Ca ratio was observed in *O*. *officinalis* (0.774) and *O*. *glumaepatula* (0.457), respectively (Table 2).

### Chlorophyll fluorescence and associated parameters

The Fv′/Fm′ is a key parameter used to assess the efficiency of photosystem II (PSII) under light conditions. This measures the capacity of the photosystem in converting the captured light energy into chemical energy (ATP and NADPH) during photosynthesis. The Fv′/Fm′ values exhibited a significant variation between the *Oryza* species studied (Table 3). The highest value of Fv′/Fm′ was observed in *O. nivara* (0.567) followed by *O. longiglumis* (0.557)*, O*. *longistaminata* (0.556) and *O*. *australiensis* (0.550)*.*

**Table 3:** Fv′/Fm′, photochemical quenching (qP), non-photochemical quenching (qN), PhiPSII and electron transport rate (ETR) as measured in the flag leaf of the cultivated and wild rice species

S. No.	*Oryza* species	Fv′/Fm′	Photochemical quenching (qP)	Non-photochemical quenching (qN)	ΦPSII	Electron transport rate (ETR)
1.	*O. alta*	0.427 ± 0.004^ghi^	0.470 ± 0.027^ab^	1.630 ± 0.053^hi^	0.180 ± 0.002^bcd^	94.6 ± 1.0^bcd^
2.	*O. australiensis*	0.550 ± 0.020^ab^	0.470 ± 0.000^cd^	2.202 ± 0.034^ab^	0.207 ± 0.009^a^	108.7 ± 4.8^a^
3.	*O. barthii*	0.541 ± 0.023^abc^	0.417 ± 0.017^bc^	1.946 ± 0.031^cde^	0.203 ± 0.006^ab^	106.6 ± 3.2^a^
4.	*O. brachyantha*	0.457 ± 0.008^fgh^	0.222 ± 0.005^i^	1.889 ± 0.040^defg^	0.104 ± 0.000^jk^	54.7 ± 0.0^jk^
5.	*O. coarctata*	0.427 ± 0.008^ghi^	0.301 ± 0.038^efgh^	2.088 ± 0.100^abcd^	0.155 ± 0.012^e^	81.3 ± 6.6^ef^
6.	*O. eichingeri*	0.343 ± 0.007^kl^	0.302 ± 0.003^efgh^	1.489 ± 0.041^i^	0.101 ± 0.004f^k^	54.9 ± 0.7^jk^
7.	*O. glaberrima*	0.468 ± 0.025^efg^	0.307 ± 0.053^defg^	1.934 ± 0.118^cdef^	0.144 ± 0.017^efgh^	75.5 ± 8.8^fgh^
8.	*O. glumaepatula*	0.319 ± 0.00^1^	0.330 ± 0.011^defg^	1.448 ± 0.018^i^	0.102 ± 0.005^k^	50.4 ± 1.4^kl^
9.	*O. grandiglumis*	0.498 ± 0.004^cdef^	0.384 ± 0.013^cd^	1.882 ± 0.044^defg^	0.181 ± 0.006^bcd^	94.9 ± 3.1^bcd^
10.	*O. granulata*	0.492 ± 0.007^def^	0.119 ± 0.012^j^	1.971 ± 0.026^bcde^	0.061 ± 0.005^l^	43.8 ± 3.8^lm^
11.	*O. latifolia*	0.416 ± 0.016^hi^	0.536 ± 0.013^a^	1.593 ± 0.026^hi^	0.199 ± 0.001^ab^	104.4 ± 0.5^ab^
12.	*O. longiglumis*	0.557 ± 0.020^ab^	0.340 ± 0.013^cdef^	1.925 ± 0.065^def^	0.162 ± 0.001^de^	86.9 ± 1.2^de^
13.	*O. longistaminata*	0.556 ± 0.013^ab^	0.469 ± 0.011^ab^	1.748 ± 0.018^efgh^	0.206 ± 0.003^a^	108.2 ± 1.4^a^
14.	*O. meridionalis*	0.368 ± 0.009^jk^	0.334 ± 0.029^def^	1.582 ± 0.022^hi^	0.122 ± 0.008^hijk^	64.2 ± 4.1^ij^
15.	*O. meyeriana*	0.504 ± 0.019^cdef^	0.105 ± 0.027^j^	2.022 ± 0.077^abcd^	0.064 ± 0.009^l^	33.8 ± 4.9^m^
16.	*O. minuta*	0.405 ± 0.024^ij^	0.299 ± 0.035^efghi^	1.667 ± 0.084^ghi^	0.117 ± 0.004^ijk^	61.2 ± 2.2^ijk^
17.	*O. nivara*	0.567 ± 0.023^a^	0.313 ± 0.036^defg^	2.212 ± 0.199^a^	0.166 ± 0.007^cde^	87.4 ± 3.6^cde^
18.	*O. officinalis*	0.512 ± 0.012^bcde^	0.464 ± 0.020^ab^	1.693 ± 0.082^fghi^	0.188 ± 0.006^abc^	98.4 ± 3.3^abc^
19.	*O. punctata*	0.444 ± 0.011^ghi^	0.250 ± 0.012^cdefg^	1.633 ± 0.005^hi^	0.126 ± 0.014^ghij^	55.7 ± 3.9^jk^
20.	*O. rhizomatis*	0.473 ± 0.018^efg^	0.307 ± 0.030^defg^	1.755 ± 0.095^efgh^	0.129 ± 0.004^fghi^	67.7 ± 2.3^ghi^
21.	*O. ridleyi*	0.362 ± 0.018^jkl^	0.136 ± 0.011^j^	2.173 ± 0.167^abc^	0.072 ± 0.002^l^	37.7 ± 0.8^hi^
22.	*O. rufipogon*	0.531 ± 0.012^abcd^	0.317 ± 0.026^defg^	1.944 ± 0.113^cde^	0.151 ± 0.004^ef^	79.5 ± 2.0^m^
23.	*O. spontanea*	0.471 ± 0.006^efg^	0.280 ± 0.012^efghi^	1.891 ± 0.019^abcde^	0.124 ± 0.007^hijk^	65.6 ± 3.3^hij^
24.	*O. sativa* ssp*. japonica*	0.427 ± 0.011^ghi^	0.347 ± 0.048^cde^	1.748 ± 0.033^efgh^	0.147 ± 0.014^efg^	77.1 ± 7.3^efg^
25.	*O. sativa*var*. javanica*	0.463 ± 0.012^fg^	0.263 ± 0.032^fghi^	1.607 ± 0.042^hi^	0.120 ± 0.007^ijk^	62.8 ± 3.6^ij^
26.	*O. sativa* ssp*. indica*	0.465 ± 0.009^efg^	0.224 ± 0.021^hi^	1.888 ± 0.049^defg^	0.121 ± 0.004^hijk^	63.4 ± 1.9^ij^

Values represent mean ± SE, n = 3 data from different plants; each data point is an average of 5 values. Mean values ± SE between rows within a column were compared for significance and different letters indicate they are statistically significant at *p* ≤ 0.05

Photochemical quenching (qP) is a measure of the efficiency with which absorbed light energy is used for photochemistry in photosynthesis. Photochemical quenching varied significantly among the *Oryza* species studied. Maximum qP was observed in *O. latifolia* (0.536) which was at par with *O*. *australiensis* (0.470), and *O*. *alta* (0.470) (Table 3).

Non-photochemical quenching (qN) refers to a mechanism used by plants to dissipate excess light energy as heat. Significant difference was observed among rice species for qN values (Table 3). Maximum qN was observed in *O*. *nivara* (2.212); followed by *O*. *australiensis* (2.202).

The ΦPSII measures the proportion of absorbed photons that are utilized by PSII for photochemical reactions, such as the splitting of water molecules and the generation of an electron transport chain that drives ATP and NADPH production. Significant variation for ΦPSII was observed among the *Oryza* species studied (Table 3). Maximum ΦPSII was observed in* O*. *australiensis* (0.207) followed by *O*. *longistaminata* (0.206)*, O*. *barthii* (0.203) and *O. latifolia* (0.199).

ETR (electron transport rate) is a critical parameter used to quantify the rate at which electrons are transferred through the photosynthetic electron transport chain in the light reactions of photosynthesis. The fraction of electron flow partitioned into photosynthesis depends on Ci, and CO_2_ compensation point which is temperature dependent. Hence, the PN would not be linearly associated with the ETR under changing Ci and temperature. The ETR values varied significantly among the *Oryza* species studied (Table 3). Maximum ETR was observed in *O*. *australiensis* (108.7) followed by *O*. *longistaminata* (108.2), *O*. *barthii* (106.6) and *O. latifolia* (104.4).

### Cluster analysis

**Fig. 1:**
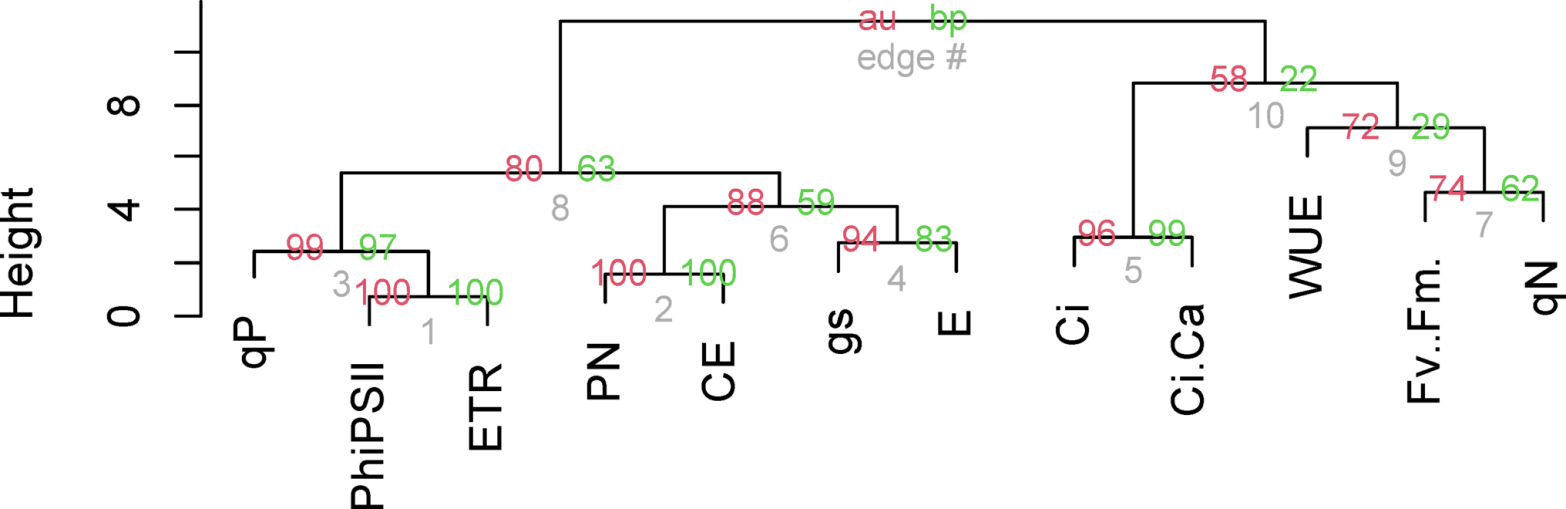
Dendrogram for 12 photosynthetic parameters using Ward’s method and Euclidean distance. AU (red) and BP (green), *p* values shown. The vertical axis (“height”), correspond to the dissimilarity/distance at which clusters merge

A dissimilarity indices-based cluster analysis was performed using all the gas exchange and chlorophyll fluorescence parameters along with its derived parameters for all species. The hierarchical clustering dendrogram (Fig. 1) grouped the 12 parameters into 2 major clusters and 5 minor clusters. Among these: Cluster I included qP, ΦPSII and ETR, Cluster II included PN and CE, Cluster III included E and gs, Cluster IV included Ci and Ci/Ca, Cluster V included qN, Fv′/Fm′ and WUE.

**Fig. 2:**
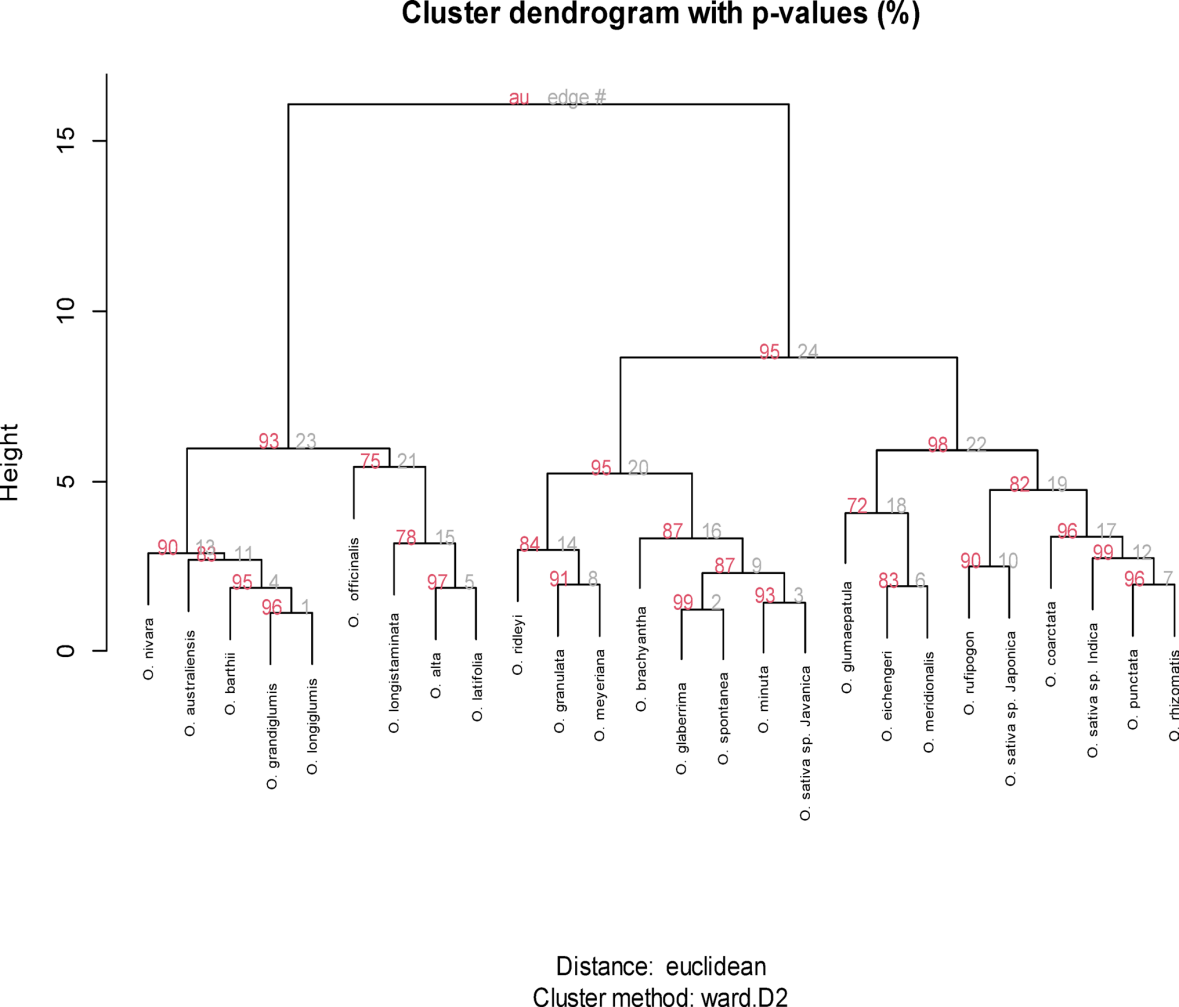
Dendrogram for 26 taxa based on all photosynthetic parameters using Ward’s method and Euclidean distance. AU (red) and *p* values (grey) shown. The vertical axis (height), correspond to the dissimilarity/distance at which clusters merge

Hierarchical cluster grouped the studied 26 taxa into 2 major clusters and 6 minor clusters as visualized in the dendrogram (Fig. 2). Cluster I included *O*. *nivara*,* O*. *australiensis*,* O*. *barthii*,* O*. *grandiglumis*, and *O*. *longiglumis*. Cluster II included* O*. *officinalis*,* O*. *longistaminata*,* O*. *alta* and* O*. *latifolia.* Cluster III included *O. ridleyi*, *O. granulata*, and *O. meyeriana*. Cluster IV included *O. brachyantha*, *O. glaberrima*, *O. spontanea*, *O. minuta*, and *O. sativa* var. *javanica.* Cluster V grouped *O. glumaepatula*, *O. eichingeri*, and *O. meridionalis.* Cluster VI grouped *O. rufipogon*, *O. sativa* subsp. *japonica*, *O. coarctata*, *O. sativa* subsp. *indica*, *O. punctata*, and *O. rhizomatis.* Cluster I and II included high-photosynthesis taxa characterized by high CE, qP and ETR. The *Oryza* species within a cluster had exhibited similar leaf photosynthetic parameters.

### Principal component analysis (PCA)

**Fig. 3:**
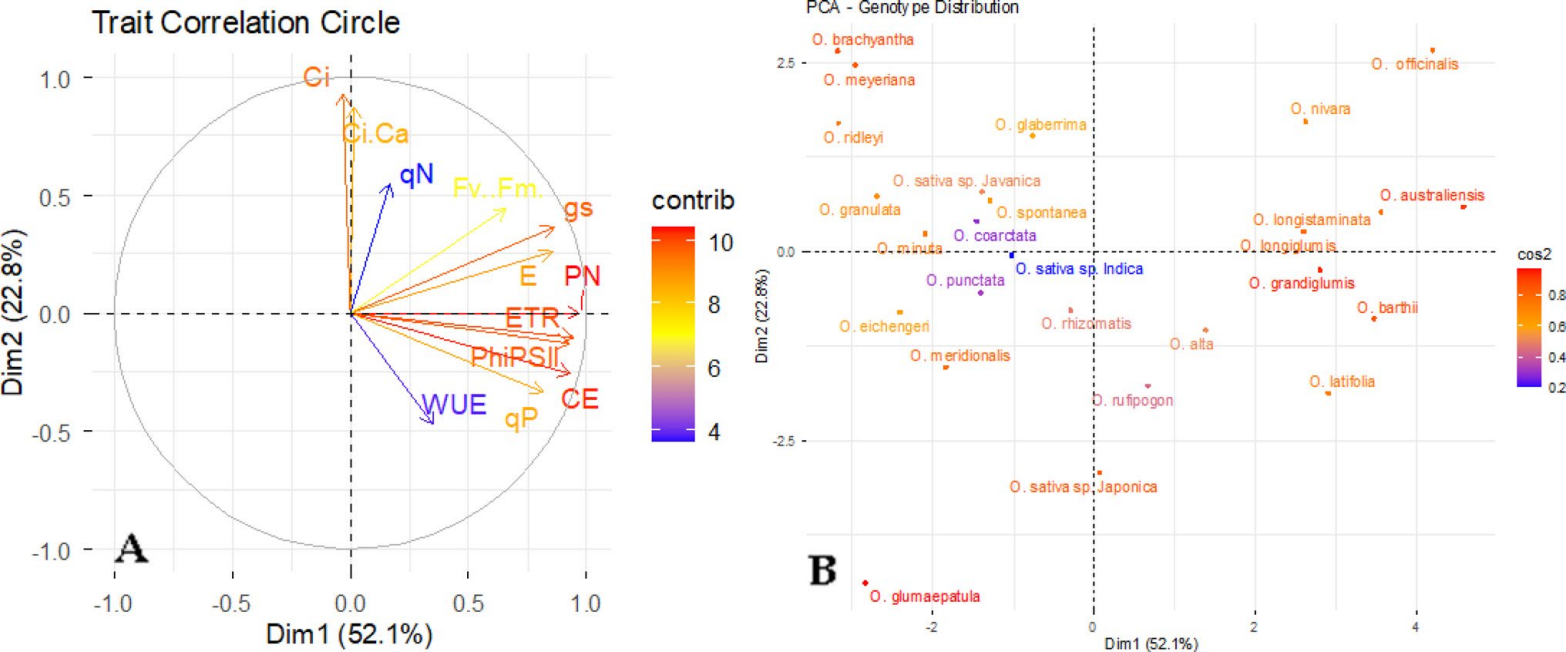
Principal component analysis biplot. **A** Photosynthetic parameters (their contribution represented in heatmap) studied in *Oryza* species. **B** *Oryza* species, studied for various photosynthetic parameters

All photosynthetic and chlorophyll fluorescence measures were used as the descriptors for PCA to explore patterns of variation and trait relationships. Figure 3 represents the PCA biplot. The biplot study underscored the level of association between different parameters and the species related to PC1 and PC2. The first two principal components (PC1 and PC2) accounted for 52.1% and 22.8% of the total variation, respectively. Together, they explained ca. 75% of the cumulative variance. Coordinate-1 was driven by PN, E, gs, Fv′/Fm′, and qN. The coordinate-2 was primarily influenced by measures such as Ci and Ci/Ca. At coordinate-3 ETR, ΦPSII, CE, qP, and WUE were clustered together. The biplot revealed clear separation among *Oryza* species. For instance, in coordinate-1, the 5 *Oryza* species i.e. *O. australiensis*, *O*. *longiglumis*, *O*. *longistaminata*, *O*. *nivara*, and *O*. *officinalis* were clustered closely together indicating similarity in their photosynthetic profiles. In coordinate-2, nine *Oryza* species i.e. *O. ridleyi*, *O. granulata*, *O. meyeriana*, *O. brachyantha*, *O. glaberrima*, *O. spontanea*, *O. minuta*, *O. coarctata*, and *O. sativa* var. *javanica* were clustered. The PC1 and PC2 were negative for 6 *Oryza* species, viz., *O. alta*, *O. barthii*, *O*. *grandiglumis*, *O*. *latifolia*, *O*. *rufipogon* and *O*. *sativa* subsp. *japonica* placed at coordinate-3. In coordinate-4, six *Oryza* species i.e. *O. glumaepatula*, *O. eichingeri*, *O. meridionalis*, *O. sativa* subsp. *indica*, *O. punctata*, and *O. rhizomatis* appeared (Fig. 3). These patterns highlight the potential of PCA in identifying genotype groups with superior photosynthetic trait. Overall, PCA helped in reducing the dimensionality of the dataset while preserving most of the variation, allowing for visualization of genotype distribution based on multivariate trait data and identification of parameters contributing most to diversity.

### Genotypic correlation analysis

A genotypic correlation analysis was performed using the photosynthetic measures across 26 taxa to assess the strength and direction of linear relationships between them (Table 4). Significant correlation (*p* ≤ 0.05 or *p* ≤ 0.01) was observed, indicating inter-dependence among physiological parameters studied. The strongest positive and significant correlation was observed between PN and CE (r = 0.9561**). The correlation of PN with gs was (r = 0.9003**), E (r = 0.8236**), Fv′/Fm′ (r = 0.6685**), ΦPSII (r = 0.841**), qP (r = 0.6879**), and ETR (r = 0.8567**). Non-significant genotypic correlation was observed between PN and Ci, Ci/Ca and qN, WUE. Photosynthetic parameters such as PN, gs, E, Fv′/Fm′, ΦPSII, qP, and ETR exhibited strong positive inter-relationships.

**Table 4:** Genotypic correlation coefficient for various photosynthetic parameters

	*PN*	*gs*	*Ci*	*E*	*ΦPSII*	*qP*	*qN*	*ETR*	*Ci/Ca*	*WUE*	*CE*
*gs*	0.9003**										
*Ci*	− 0.0356	0.3406									
*E*	0.8236**	0.8481**	0.1543								
*PhiPSII*	0.841**	0.7077**	− 0.1013	0.7778**							
*qP*	0.6879**	0.5688**	− 0.2839	0.7068**	0.9044**						
*qN*	0.2206	0.2253	0.3879	0.125	0.063	− 0.3287					
*ETR*	0.8567**	0.7229**	0.1051	0.805**	0.9929**	0.8932**	0.105				
*Ci/Ca*	− 0.0035	0.3313	0.819**	0.1952	− 0.0541	− 0.1565	0.2482	− 0.0439			
*WUE*	0.3372	0.1489	− 0.3128	− 0.2218	0.2028	0.1315	− 0.0269	0.1743	− 0.3059		
*CE*	0.9561**	0.7408**	− 0.3128	0.7365**	0.8369**	0.7267**	0.1337	0.8532**	− 0.2308	0.3961*	
Fv′/Fm′	0.6685**	0.6837**	0.3047	0.5874**	0.5231**	0.1821	0.5739**	0.557**	0.2632	0.1076	0.5757**

For statistical significance, * indicate significance at *p* ≤ 0.05; ** indicate significance at *p* ≤ 0.01

### Path analysis

Path analysis was performed to compartmentalize the correlation values into the direct and indirect effects of associated photosynthetic parameters with PN as dependent variable (Table 5). Highly significant positive correlations were observed between PN and the following measures CE (r = 0.9561**) followed by gs (r = 0.9003**), ETR (r = 0.8567**), ΦPSII (r = 0.841**), E (r = 0.8236**), qP (r = 0.6879**) and Fv′/Fm′ (r = 0.6685**). These results suggest that these parameters are key determinants of photosynthetic performance. In contrast, some parameters showed non-significant correlations with PN: Ci (r = − 0.0356 NS), qN (r = 2206 NS), WUE (r = 0.3372 NS), Ci/Ca (r = − 0.0035 NS). The residual effect was 0.368, indicating that approximately 63% of the variation attributed to PN was explained through the measures studied, warranting for incorporation of additional parameters or measures that quantify photosynthesis.

**Table 5:** Path analysis (using genotypic correlation matrix) for various photosynthetic parameters

Trait	*gs*	*Ci*	*E*	*ΦPSII*	*qP*	*qN*	*ETR*	*Ci/Ca*	*WUE*	*CE*	*Fv'/Fm'*	*PN*
*Gs*	0.079765	0.085896	0.087826	− 0.08239	0.347342	0.04189	− 0.3807	− 0.01207	0.015199	0.639315	0.078266	0.9003**
*Ci*	0.027166	0.252206	0.015983	0.011799	− 0.17335	0.072126	0.055374	− 0.02985	− 0.03193	− 0.26998	0.034882	− 0.0356
*E*	0.06765	0.038925	0.103554	− 0.09055	0.431666	0.023244	− 0.42397	− 0.00711	− 0.02264	0.635604	0.067244	0.8236**
*ΦPSII*	0.056451	− 0.02556	0.080543	− 0.11642	0.552308	0.011723	− 0.52291	0.001971	0.020701	0.722267	0.059886	0.841**
*qP*	0.045368	− 0.07159	0.073197	− 0.10529	0.61069	− 0.06112	− 0.47043	0.005703	0.013421	0.627129	0.02084	0.6879**
*qN*	0.01797	0.097833	0.012945	− 0.00734	− 0.20075	0.185936	− 0.05531	− 0.00905	− 0.00274	0.115401	0.065696	0.2206
*ETR*	0.057658	− 0.02652	0.08336	− 0.11559	0.545475	0.019525	− 0.52667	0.001599	0.017796	0.736308	0.063764	0.8567**
*Ci/Ca*	0.026424	0.206546	0.020217	0.006296	− 0.09556	0.046155	0.023105	− 0.03644	− 0.03123	− 0.19917	0.030134	− 0.0035
*WUE*	0.011876	− 0.07888	− 0.02297	− 0.02361	0.080294	− 0.005	− 0.09182	0.011149	0.102079	0.341802	0.012313	0.3372
*CE*	0.05909	− 0.0789	0.076268	− 0.09743	0.443776	0.024863	− 0.44935	0.008411	0.040429	0.863006	0.065901	0.9561**
Fv′/Fm′	0.054534	0.07685	0.060829	− 0.0609	0.111176	0.106705	− 0.29336	− 0.00959	0.01098	0.496806	0.114476	0.6685**

For statistical significance, * indicate significance at *p* ≤ 0.05; ** indicate significance at *p* ≤ 0.01

### Multivariate comparison of photosynthetic parameters

**Fig. 4:**
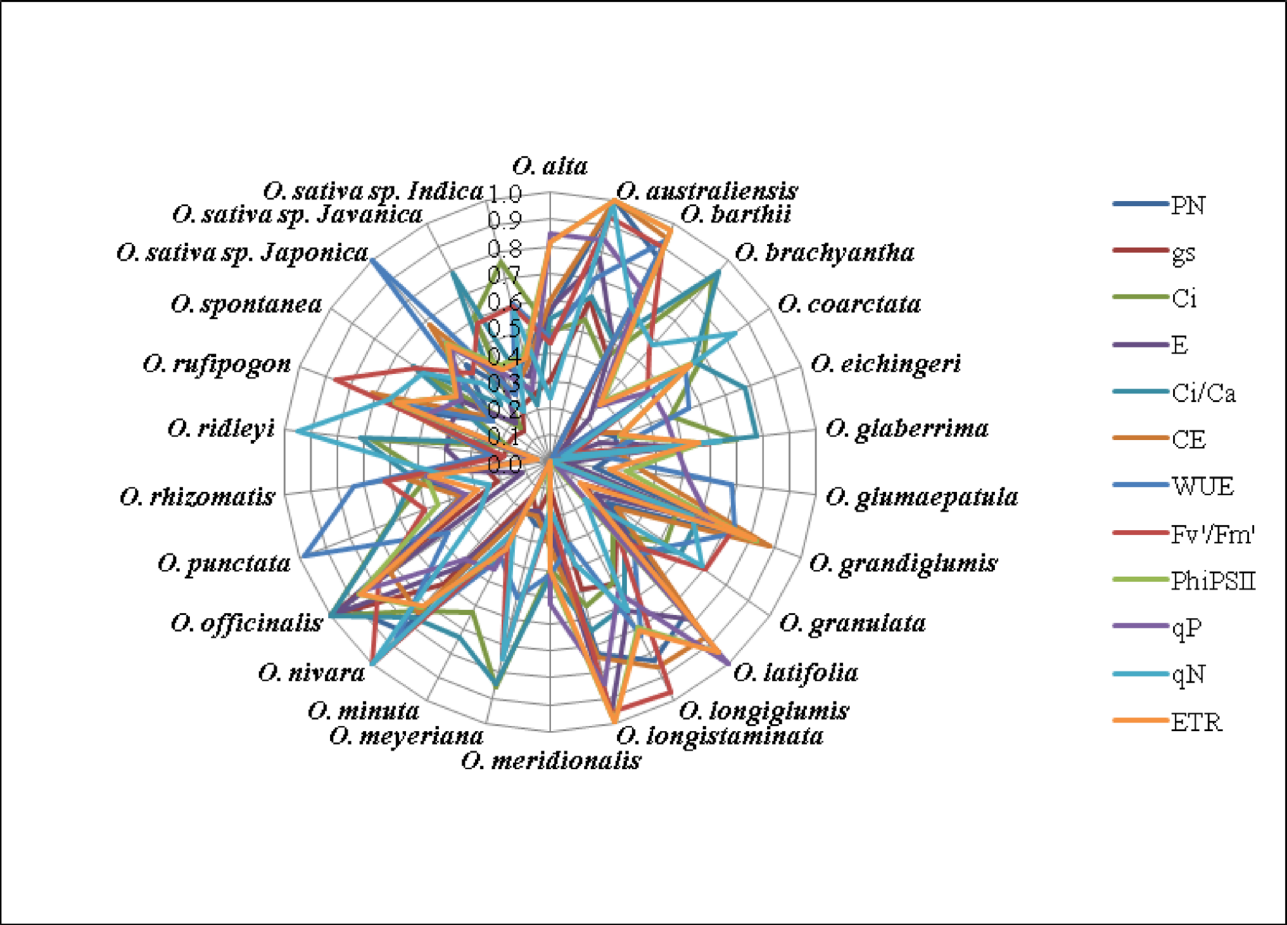
Radar plot for photosynthetic parameters studied in *Oryza* species. Axes correspond to measured physiological traits, and polygon shapes show the relative performance of each species across traits. Values were normalized to enable comparison across variables with different units

To visualize multivariate trait variation among species, radar plots were constructed using normalized values of the studied photosynthetic parameters (Fig. 4). Additionally, MANOVA and PERMANOVA analysis also exhibited the significance for the studied parameters across the wild rice taxa (Supplementaryfile.docx, Tables S4 and S5). Radar plot reveals substantial inter-specific variation in gas exchange parameters and chlorophyll fluorescence traits across cultivated and wild *Oryza* species. Parameters with different units and scales were min–max normalized to enable fair comparison. PN, gs, and E varied widely, with species such as *O*. *australiensis, O*. *officinalis, O*. *longiglumis*,* O*. *latifolia, O*. *longistaminata*, *O*. *barthii*,* O*. *grandiglumis*, *and O*. *nivara* exhibited broader and more uniform radar shapes, indicating consistent high performance across the photosynthetic parameters studied. Certain trade-offs were evident; for example, species with higher Ci values tend to show moderate PN and lower WUE, suggesting potential biochemical limitations in internal CO_2_ fixation or Rubisco activity.

### Transcriptome analysis

**Fig. 5:**
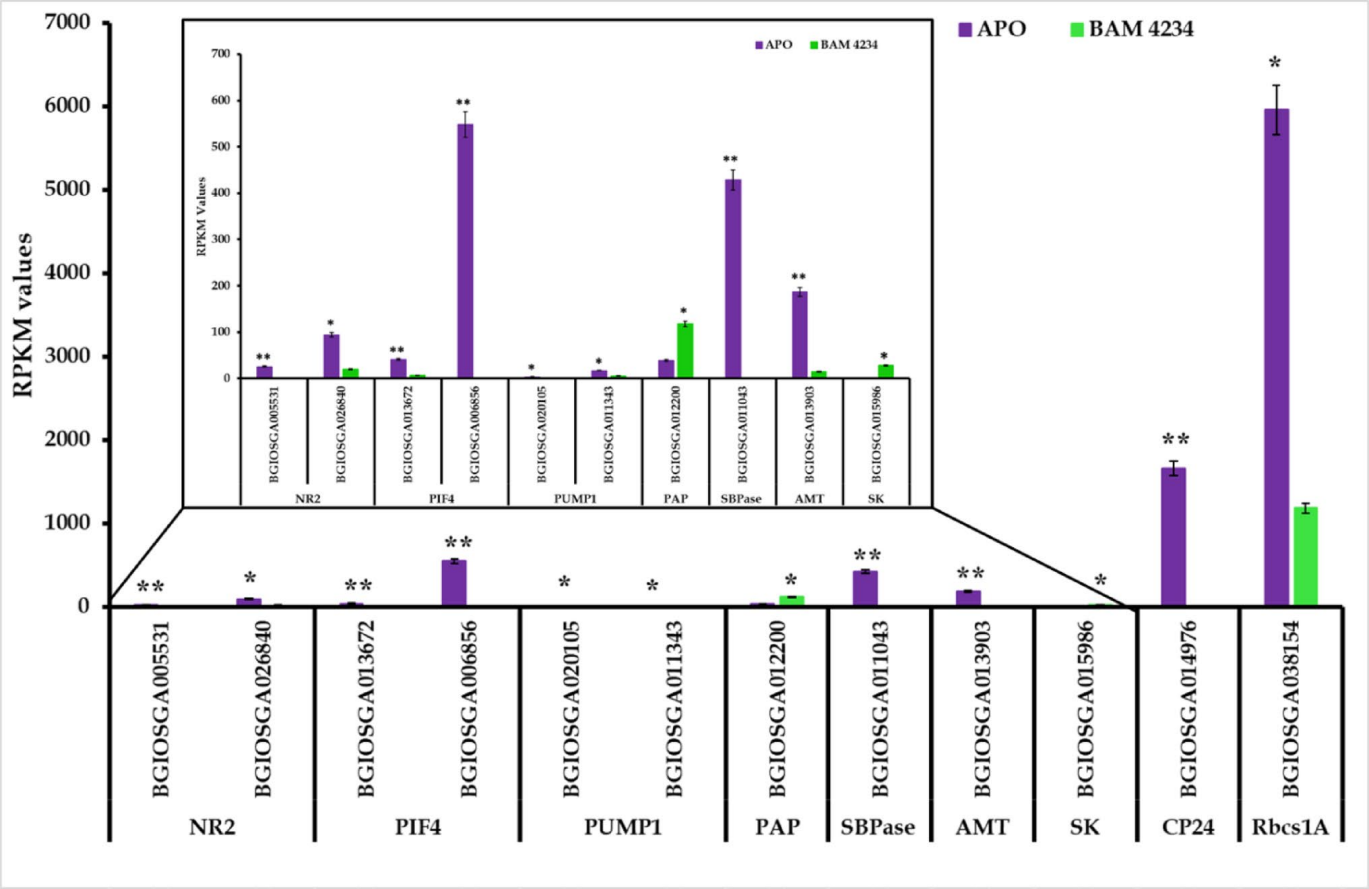
Gene expression profiles (in RPKM values, Y-axis) for the nine genes (12 transcripts, X-axis) as depicted in bar graph for the compared genotypes, APO and BAM4234. Inset: Of these nine genes, first seven were given as inset since their RPKM values were less than 1000. Significance for each of the transcript’ expression levels between APO and BAM4234 denoted as **FDR cut-off at *p* ≤ 0.01; *FDR cut-off at *p* ≤ 0.05

Differential gene expression analysis between APO and BAM4234 had revealed the set of statistically significant genes (at false-discovery rate cut-off 0.05) that were differentially regulated between the studied genotypes (SupplementaryfileTome.xlsx). Of the significantly differentially regulated genes, nine (12 transcripts) were most prominent and associated with photosynthesis traits were found to be *SBPase* (BGIOSGA011043), *PIF4* (BGIOSGA013672, BGIOSGA006856), *NR2* (BGIOSGA005531, BGIOSGA026840), *PAP* (BGIOSGA012200), *AMT* (BGIOSGA013903)**,** *PUMP* (BGIOSGA020105, BGIOSGA011343), *SK* (BGIOSGA015986), *CP24* (BGIOSGA014976), and *RbcS1A* (BGIOSGA038154) (Fig. 5). Results demonstrated that in APO genotype, *SBPase*, *PIF4*, *NR2*, *AMTs*, *PUMP*, *CP24*, and *RbcS1A* were significantly upregulated, while *PAP* and *SK* were significantly downregulated.

## Discussion

Genetic improvement in rice through enhancing harvest index got saturated; hence, increase in total biomass production through improving photosynthesis traits is the key to further increasing yields (van Bezouw et al. 2019; Furbank et al. 2019; Acevedo-Siaca et al. 2021). Natural genetic variability for photosynthesis traits especially from wild genetic resources, CWRs, is a promising yet underutilized resource for breeding crops with improved photosynthetic capacity leading to higher yields (Long et al. 2015; Fernie and Yan 2019; Khan et al. 2020; Jathar et al. 2022). The natural variability for photosynthesis traits among the rice wild relatives was studied through gas exchange and chlorophyll fluorescence parameters across 26 *Oryza* taxa and is reported here.

### Gas exchange and associated parameters

Based on the PN, gs, and Ci measures studied across 26 taxa infers the underlying biochemical limitations that affect carbon assimilation. Wild taxa *O. brachyantha* showed the lowest PN (5.2) despite very high Ci (312.9) among all taxa studied (Table 1). Source-sink dynamics is a major determinant of the yield and productivity of rice (Rosado-Souza et al. 2023). Targeting source and sink strength for crop yield increase requires a comprehensive genetic and metabolic understanding of desirable source and sink features (Singh et al. 2025). This preliminary observation warrants for a detailed study on the phosphate levels and the expression pattern for the genes or its products involved in sucrose and starch synthesis in *O. brachyantha*. Additionally, higher Ci values despite lesser gs values (0.103) might possibly be due to higher photorespiration (Sharkey 2016). Thus, this species is biochemically limited (or regulated), especially at the level of phosphate limiting or RuBP regeneration**.** The PN, Ci and gs in *O. glumaepatula* was 8.7, 188.7, and 0.068, respectively (Table 1)**.** This species exhibited lowest Ci and gs which may be possibly due to restricted CO_2_ entry indicating a strong Rubisco-limited photosynthetic response. O*. australiensis* stands out as the most physiologically efficient species with PN (26.0), Ci (263.3) and gs (0.338), balancing gas exchange and biochemical capacity (Table 1). Thus, this species exhibited balanced Rubisco carboxylation and RuBP regeneration with highest photosynthetic rates among the taxa studied and our results corroborate with Mathan et al. (2021a). Faster leaf elongation rates and enhanced photosynthetic biochemistry in *O. australiensis* was well documented (Scafaro et al. 2016; Chatterjee et al. 2016; Mathan et al. 2021a; Phillips et al. 2022). Therefore, *O. australiensis* is a potential source for rice improvement through improving photosynthesis. Although *O. officinalis* exhibited the highest gs (0.507) and Ci (325.9), its PN (24.7) was found to be on par with *O. australiensis* (Table 1), while highest PN is expected. Possibly, similar to *O. brachyantha*, assimilation might be inhibited (mildly, not to the extent as in *O. brachyantha*) reflecting higher Ci values and lesser PN. Improving the sucrose or starch synthesis to match with assimilation rate would potentially enhance PN higher than *O. australiensis*.

The PN, Ci and gs values for *O. sativa* subsp. *japonica* were 15.2, 211.4, 0.135, respectively (Table 1). It had more Ci as compared to *O. glumaepatula* and low PN in comparison to *O. australiensis* and *O. officinalis*. This clearly represents a RuBP-regeneration limited response. The Farquhar, von Caemmerer, and Berry (FvCB) model classifies primary biochemical limitations as Rubisco limited (low Ci, low PN) or RuBP or TPU limited (high Ci, low PN) and emphasizes the importance of both stomatal and non-stomatal factors in species performance (Farquhar et al. 1980; Sharkey 1985). Overlaying our results on this model, the taxa can be grouped into two classes. One with low Ci and low PN **(**Rubisco limited) and another with high Ci and low PN (RuBP or TPU limited). These results highlight the importance of differentiating between stomatal and biochemical constraints when selecting species for high photosynthetic efficiency. Further analysis of Rubisco content, enzyme kinetics, and electron transport capacity in wild species can help in pinpointing specific biochemical bottlenecks. To further elucidate the physiological mechanisms underlying the observed species variation in photosynthetic performance, future studies should include detailed A-Ci (CO_2_ response) and A-Q (light response) curves. These measurements will allow a more precise separation of stomatal vs. biochemical limitations and quantification of key photosynthetic parameters.

Stomatal traits play a critical role in regulating gas exchange and water use efficiency, and thereby directly influence photosynthesis. In the present study, we observed variability in photosynthetic and chlorophyll fluorescence parameters among wild rice accessions, which may be partially attributed to underlying stomatal characteristics. Although stomatal density was not directly measured in this study, the findings of Chatterjee et al. (2020) imply that accessions with higher PN, CE, and gs could possess more favorable stomatal traits (e.g., optimal density and size), enable efficient CO_2_ uptake while minimize water loss. Future work involving stomatal imaging and quantification could clarify this relationship and help in identifying physiological markers in wild relatives of rice.

### Chlorophyll fluorescence and associated parameters

Chlorophyll fluorescence is a valuable, non-invasive tool for understanding the efficiency of photosystem II and overall photosynthetic performance (Tsai et al. 2019; Guidi et al. 2019; Jumrani et al. 2024a, b). Measuring chlorophyll fluorescence parameters such as qP, qN, ΦPSII and ETR, helps to identify genotypes with superior light energy conversion for an efficient photosynthesis (McAusland et al. 2019; Herritt et al. 2020). The intricate relationships between chlorophyll fluorescence kinetics and photosynthesis contribute to our understanding of the biophysical processes underlying photosynthesis (Sommer et al. 2023). The comparative analysis of ETR, qP, and PN across 26 *Oryza* taxa reveals distinct physiological differences between wild and cultivated rice. Some wild taxa such as *O. australiensis*, *O. longistaminata*, *O. latifolia, O. barthii,* and* O*. *officinalis* consistently exhibited higher values for ETR (> 95), qP (> 0.4), and PN (> 20) (Tables 1 and 3). This correlates with efficient photochemical energy conversion and carbon assimilation.

It was also evident from our results that highest value for Fv′/Fm′ and qN was observed in *O. nivara* (0.567 and 2.21) and *O. australiensis* (0.555 and 2.20) highlighting their strong photoprotective capacity and efficient conversion of absorbed light energy to chemical energy (Table 3). This mechanism is critical for preventing photodamage to photosystem II under high or fluctuating light intensities. Similarly, a high Fv′/Fm′ suggests that both *O. nivara* and *O. australiensis* maintain a high maximum quantum efficiency of PSII photochemistry even under illuminated conditions, reflecting a superior ability to utilize absorbed light for photosynthetic electron transport. These findings suggest that *O. nivara* and *O. australiensis* may contain alleles that enhance photoprotection and photosynthesis or may share adaptive traits for light stress tolerance.

### Association studies for photosynthetic traits

Gas exchange and chlorophyll fluorescence measures were subjected to association studies using correlation and path analysis. PN exhibited a positive correlation with gs, E, CE, qP, Fv′/Fm′, ETR, and ΦPSII. The genotypic correlation and path analysis results (Tables 4 and 5) helped in identifying five best parameters viz., CE, gs, Fv′/Fm′, qP, and ETR that are closely associated with photosynthetic performance. Out of these five, CE and qP were found to be the most important measure that can be used in selection process, in breeding for photosynthetic improvement. Understanding the genetics behind the physiology of CE and qP will fetch major advantages in the scientific intervention to improve photosynthesis for enhanced productivity. The species that had exhibited better qP were *O*. *australiensis*,* O*. *officinalis, O. longistaminata*, and *O. latifolia*. Similarly, the species that had exhibited better CE were* O*. *australiensis*,* O*. *officinalis*,* O. longiglumis*, *O. grandiglumis*, *O*. *nivara*, and* O*. *barthii*. Notably, *O. australiensis* and *O. officinalis* were common for both qP and CE, exhibiting better values for both these measures. Among the rice wild relatives studied these two species will be the preferred starting point to understand the genetics underlying the physiology for a better qP and CE. Interestingly, highest value for qP was recorded by *O. latifolia* (Table 3), proving its efficiency in chlorophyll fluorescence (light reaction) part of photosynthesis. However, its gas exchange values suggest that the assimilation is mainly limited due to lesser gs values that limit the availability of the CO_2_ supply. This can be validated through modulating the stomata of *O. latifolia* for a higher CO_2_ supply to confirm whether such modifications lead to a higher assimilation than the unmodified ones. Besides *O. latifolia*, *O. alta* and *O. grandiglumis* were the two other taxa studied from the CCDD genome members. Of these, *O. alta* had a qP value at par with *O. latifolia* and *O. grandiglumis* had CE value at par with *O. latifolia.* (Tables 2 and 3). *O. grandiglumis* exhibited a higher CE in spite of lower qP value which suggests, that *O. grandiglumis* is efficient in gas exchange part (dark reaction) of photosynthesis. Development of successful inter-specific hybrids between *O. latifolia* and *O. grandiglumis* might yield progenies with efficiency in both light and dark reaction parts of photosynthesis and hence higher biomass. Among the wild relatives that had led to domestication events, *O*. *nivara* and *O. rufipogon* both had similar qP values but *O*. *nivara* had significantly higher PN.

The clustering pattern in the dendrogram (Fig. 2) also grouped the accessions that can be associated with the variability in the photosynthesis efficiency. The species *O*. *australiensis*,* O*. *officinalis, O*. *nivara,* and* O*. *barthii* that had exhibited higher photosynthetic efficiency among the studied accessions were grouped into one cluster. Other wild species which also have shown higher PN and associated measures were *O. grandiglumis*, *O. longiglumis, O. latifolia,* and* O*. *longistaminata*.

### Transcriptome analysis:

The key significantly differentially regulated genes associated with photosynthesis trait were identified (Fig. 5 and SupplementaryfileTome.xlsx) using the flag-leaf transcriptome dataset for the highly efficient photosynthesis genotype APO, and the normal genotype BAM4234. Sedoheptulose 1, 7 bisphosphatase (*SBPase*) is involved in ribulose 1, 5 bisphosphate (RuBP) regeneration and thereby regulates photosynthetic carbon fixation, and a key regulatory point for Calvin-Benson cycle (Ding et al. 2016; Meloni et al. 2023). Hence higher expression levels of this gene in APO is correlative with highly efficient photosynthesis performance (Fig. 5). Phytochrome Interacting Factor 4 (*PIF4*) is a transcription factor well-known to regulate growth and photosynthesis through balancing chlorophyll biosynthesis (Song et al. 2014; Legris et al. 2016). Nitrate Reductase 2 (*NR2*) is a key gene involved in nitrogen assimilation, a pre-requirement for biosynthesis of the Rubisco protein—the most abundant protein on earth (Guilherme et al. 2019; Krämer et al. 2022). Upregulation of this gene is positively correlated with higher Rubisco content and in turn higher carbon fixation. Ammonium transporters (*AMTs*) facilitate the movement of ammonium (NH4^+^) across cellular membranes and a key gene for nitrogen supply at appropriate space and time (Neuhäuser et al. 2007; Masclaux et al. 2010). The plant uncoupling mitochondrial proteins (*PUMPs*) gene product specifically modulate photosynthesis by influencing cellular energy balance and mitigating oxidative stress (Lima et al. 2022). Shikimate kinase (*SK*) gene were known to play a central role in shikimate pathway. It balances the carbon allocation between the secondary (specialized) metabolite biosynthesis and the primary metabolite biosynthesis (Yuan et al. 2022). Chlorophyll a/b—binding protein gene (*CP24*) codes for chlorophyll proteins. These proteins act as a non-photochemical quencher, to prevent photoinhibition (Bassi et al. 1997; Rong et al. 2025). Ribulose 1,5-bisphosphate carboxylase/oxygenase small subunit 1A (*Rbcs1A*) isoform is known to act in additive fashion with its paralogs in the genome for Rubisco biosynthesis for efficient carbon fixation. Reduced expression levels of *RbcS* are known to be linked with proportionate reduction in the Rubisco protein content, and is correlative with net assimilation rate (Izumi et al. 2012).

## Conclusion

The findings of this study from 26 *Oryza* taxa underscores a key message that the cultivated species (*O. sativa* and *O. glaberrima*) had exhibited a narrowed photosynthetic range as compared to their wild relatives. Reintroducing the photosynthetically valuable alleles from wild *Oryza* species into cultivars may exhibit robust photosynthetic machinery coupled with higher yield since the species within primary gene pool are inter-crossable. Although rice wild relatives from secondary and tertiary gene pool also do fertilize successfully, might require embryo rescue techniques to revive and regenerate successfully the newly formed hybrids. An efficient photosynthetic system found in *O. australiensis*,* O*. *officinalis, O*. *nivara,* and* O*. *barthii* underscores its potential for studying the genetics of physiological traits with special reference to photosynthesis, besides being candidates for crop improvement through breeding approaches. Additionally, those species that performed better only for light reaction (*O*. *australiensis*,* O*. *officinalis, O. longistaminata*, and *O. latifolia)*, besides these species those were good for only dark reaction (*O. longiglumis*, *O. grandiglumis*, *O*. *nivara*, and *O. barthii*) might be used to introgress and generate derivatives exhibiting better for both light and dark reaction and thereby improve their photosynthetic machinery. We compared the gene expression profiles between the photosynthetically high efficient genotype APO, with a normal genotype BAM4234 using flag-leaf transcriptome dataset, generated by us and reported recently (Rangan et al. 2025). Our results underscored the key genes associated to improve photosynthesis that were significantly upregulated in APO genotype. Notable ones were *CP24*, *Rbcs1A*, *NR2*, *PIF4*, *PUMP1*, *SBPase*, and *AMT* (Fig. 5). These require detailed expressive and functional validation in wild rice species to identify allelic forms exhibiting high photosynthetic efficiency for its potential use in crop improvement.

## Supplementary Information

Below is the link to the electronic supplementary material.

Supplementary Material 1

Supplementary Material 2

## Data Availability

The raw reads of the transcriptome dataset used in this study was generated by us and is publicly available for access through the accession number E-MTAB-8361. Among these datasets, only flag leaf data was studied with reference to photosynthesis traits. The supporting data pertaining to this study is provided within this manuscript and the additional information was given as supplementary information (Suplementaryfile.docx; SupplementaryfileTome.xlsx).

## References

[CR1] Acevedo-Siaca LG, Coe R, Wang Y, Kromdijk J, Quick WP, Long SP (2020) Variation in photosynthetic induction between rice acces sions and its potential for improving productivity. New Phytol 227:1097–1108. 10.1111/nph.16454 32124982 10.1111/nph.16454 PMC7383871

[CR2] Acevedo-Siaca LG, Dionora J, Laza R, Paul Quick W, Long SP (2021) Dynamics of photosynthetic induction and relaxation within the canopy of rice and two wild relatives. Food Energy Secur 10(3):e286. 10.1002/fes3.286 34594547 10.1002/fes3.286 PMC8459282

[CR3] Bassi R, Sandona D, Croce R (1997) Novel aspects of chlorophyll a/b-binding proteins. Physiol Plantarum 100:769–779. 10.1111/j.1399-3054.1997.tb00004.x

[CR4] Blighe K (2021) PCA tools: principal component analysis tools. R package version 2.14.0. URL: https://bioconductor.org/packages/PCAtools/

[CR5] Brar DS, Khush GS (2018) Wild relatives of rice: a valuable genetic resource for genomics and breeding research. In: Mondal T, Henry R (eds) The wild oryza genomes. Compendium of plant genomes. Springer, Cham. 10.1007/978-3-319-71997-9_1

[CR6] Burgess AJ, Masclaux-Daubresse C, Strittmatter G, Weber APM, Taylor SH, Harbinson J et al (2023) Improving crop yield potential: underlying biological processes and future prospects. Food Energy Secur 12:e435. 10.1002/fes3.435 37035025 10.1002/fes3.435 PMC10078444

[CR7] Chatterjee J, Dionora J, Elmido-Mabilangan A, Wanchana S, Thakur V, Bandyopadhyay A et al (2016) The evolutionary basis of naturally diverse rice leaves anatomy. PLoS ONE 11:e0164532. 10.1371/journal.pone.0164532 27792743 10.1371/journal.pone.0164532 PMC5085062

[CR8] Chatterjee J, Thakur V, Nepomuceno R et al (2020) Natural diversity in stomatal features of cultivated and wild *Oryza* species. Rice 58:1–20. 10.1186/s12284-020-00417-0 10.1186/s12284-020-00417-0 PMC7441136 32816163

[CR9] de Mendiburu, F (2021) agricolae*:* statistical procedures for agricultural research (v1.3-5) (https://CRAN.R-project.org/package=agricolae)

[CR10] Ding F, Wang M, Zhang S, Ai X (2016) Changes in SBPase activity influence photosynthetic capacity, growth, and tolerance to chilling stress in transgenic tomato plants. Sci Rep 6:32741. 10.1038/srep32741 27586456 10.1038/srep32741 PMC5009361

[CR11] FAO (2024) Rice production, utilization and stocks. https://www.fao.org

[CR12] Farquhar GD, von Caemmerer S, Berry JA (1980) A biochemical model of photosynthetic CO_2_ assimilation in leaves of C3 species. Planta 149:78–90. 10.1007/BF00386231 24306196 10.1007/BF00386231

[CR13] Fernie AR, Yan J (2019) De novo domestication: an alternative route toward new crops for the future. Mol Plant 12:615–631. 10.1016/j.molp.2019.03.016 30999078 10.1016/j.molp.2019.03.016

[CR14] Flügge UI, Westhoff P, Leister D (2016) Recent advances in understanding photosynthesis. F1000Res 5:2890. 10.12688/f1000research.9744.1 28105322 10.12688/f1000research.9744.1 PMC5224682

[CR15] Fornasiero A, Feng T, Al-Bader N, Alsantely A, Mussurova S, Hoang NV, Misra G, Zhou Y, Fabbian L, Mohammed N, Rivera Serna L, Thimma M, Llaca V, Parakkal P, Kudrna D, Copetti D, Rajasekar S, Lee S, Talag J, Sobel-Sorenson C, Panaud O, McNally KL, Zhang J, Zuccolo A, Schranz ME, Wing RA (2024) *Oryza* genome evolution through a tetraploid lens. bioRxiv. 10.1038/s41588-025-02183-539554163

[CR16] Furbank RT, Jimenez-Berni JA, George-Jaeggli B, Potgieter AB, Deery DM (2019) Field crop phenomics: enabling breeding for radiation use efficiency and biomass in cereal crops. New Phytol 223:1714–1727. 10.1111/nph.15817 30937909 10.1111/nph.15817

[CR17] Furbank RT, Sharwood R, Estavillo GM, Silva-Perez V, Condon AG (2020) Photons to food: genetic improvement of cereal crop photosynthesis. J Exp Bot 71:2226–2238. 10.1111/nph.15817 32083680 10.1093/jxb/eraa077 PMC7135014

[CR18] Garcia A, Gaju O, Bowerman AF, Buck SA, John R, Furbank RT, Gilliham M, Millar AH, Pogson BJ et al (2023) Enhancing crop yields through improvements in the efficiency of photosynthesis and respiration. New Phytol 237:60–77. 10.1111/nph.18545 36251512 10.1111/nph.18545 PMC10100352

[CR19] Giuliani R, Koteyeva N, Voznesenskaya E, Evans MA, Cousins AB, Edwards GE (2013) Coordination of leaf photosynthesis, transpiration, and structural traits in rice and wild relatives (genus *Oryza*). Plant Physiol 162:1632–1651. 10.1104/pp.113.217497 23669746 10.1104/pp.113.217497 PMC3707562

[CR20] Guidi L, Lo Piccolo E, Landi M (2019) Chlorophyll fluorescence, photoinhibition and abiotic stress: Does it make any difference the fact to be a C3 or C4 species? Front Plant Sci 10:174. 10.3389/fpls.2019.00174 30838014 10.3389/fpls.2019.00174 PMC6382737

[CR21] Guilherme EA, Carvalho FEL, Daloso DM, Silveira JAG (2019) Increase in assimilatory nitrate reduction and photorespiration enhances CO_2_ assimilation under high light-induced photoinhibition in cotton. Environ Exp Bot 159:66–74. 10.1016/j.envexpbot.2018.12.012

[CR22] Haoran W, Guoqing C, Guozhong F (2025) Expanding viral diversity in rice fields by next-generation sequencing. Rice Sci 32:44–51. 10.1016/j.rsci.2024.12.004

[CR23] Herritt MT, Pauli D, Mockler TC et al (2020) Chlorophyll fluorescence imaging captures photochemical efficiency of grain sorghum (*Sorghum bicolor*) in a field setting. Plant Methods 16:109. 10.1186/s13007-020-00650-0 32793296 10.1186/s13007-020-00650-0 PMC7419188

[CR24] Hunter MC, Smith RG, Schipanski ME, Atwood LW, Mortensen DA (2017) Agriculture in 2050: recalibrating targets for sustainable intensification. Bioscience 67:386–391. 10.12691/jfs-13-4-1

[CR25] Izumi M, Tsunoda H, Suzuki Y, Makino A, Ishida H (2012) Rbcs1A and Rbcs3B, two major members within the *Arabidopsis* RBCS multigene family, function to yield sufficient Rubisco content for leaf photosynthetic capacity. J Exp Bot 63:2159–2170. 10.1093/jxb/err434 22223809 10.1093/jxb/err434 PMC3295403

[CR26] Jari-Oksanen F, Blanchet G, Friendly M, Kindt R, Legendre P, McGlinn D (2019) *vegan:* community ecology package. R Package Version 2.5-6. Available online at: http://CRAN.R-project.org/package=vegan

[CR27] Jathar V, Saini K, Chauhan A, Rani R, Ichihashi Y, Ranjan A (2022) Spatial control of cell division by GA-OsGRF7/8 module in a leaf explaining the leaf length variation between cultivated and wild rice. New Phytol 234:867–883. 10.1111/nph.18029 35152411 10.1111/nph.18029

[CR28] Jumrani K, Bhatia VS, Hussain S, Kataria S, Yang X, Brestic M (2024a) Effect of shading on leaf anatomical structure, photosynthesis characteristics and chlorophyll fluorescence of soybean (*Glycine max*). J Agron Crop Sci 210:12783. 10.1111/jac.12783

[CR29] Jumrani K, Bhatia VS, Kataria S, Rastogi A (2024b) The interactive effect of high temperature and water deficit stress on nitrogen fixation, photosynthesis, chlorophyll fluorescence, seed yield and quality in soybean (*Glycine max*). Plant Physiol Rep 29:125–140. 10.1007/s40502-023-00763-3

[CR30] Khan AW, Garg V, Roorkiwal M, Golicz AA, Edwards D, Varshney RK (2020) Super-pangenome by integrating the wild side of a species for accelerated crop improvement. Trends Plant Sci 25:148–158. 10.1016/j.tplants.2019.10.012 31787539 10.1016/j.tplants.2019.10.012 PMC6988109

[CR31] Kiran TV, Rao YV, Subrahmanyam D, Rani NS, Bhadana VP, Rao PR, Voleti SR (2013) Variation in leaf photosynthetic characteristics in wild rice species. Photosynthetica 51:350–358. 10.1007/s11099-013-0032-3

[CR32] Kondamudi R, Swamy KN, Rao YV, Kiran TV, Suman K, Rao DS, Rao PR, Subrahmanyam D, Sarla N, Ramana BK, Voleti SR (2016) Gas exchange, carbon balance and stomatal traits in wild and cultivated rice (*Oryza sativa* L.) genotypes. Acta Physiol Plant 38:1–9. 10.1007/s11738-016-2173-z

[CR33] Krämer K, Brock J, Heyer AG (2022) Interaction of nitrate assimilation and photorespiration at elevated CO_2_. Front Plant Sci 13:897924. 10.3389/fpls.2022.897924 35845694 10.3389/fpls.2022.897924 PMC9284316

[CR34] Legris M, Klose C, Burgie ES, Rojas CC, Neme M, Hiltbrunner A, Wigge PA, Schäfer E, Vierstra RD, Casal JJ (2016) Phytochrome B integrates light and temperature signals in *Arabidopsis*. Science 354:897–900. 10.1126/science.aaf5656 27789798 10.1126/science.aaf5656

[CR35] Li Y, Xiao JH, Chen LL, Huang XH, ChengZK HB, Zhang QF, Wu CY (2018) Rice functional genomics research: Past decade and future. Mol Plant 11:359–380. 10.1016/j.molp.2018.01.007 29409893 10.1016/j.molp.2018.01.007

[CR36] Lima RPM, Nunes-Laitz AV, Arcuri MLC, Campos FG, Joca TAC, Monteiro GC, Kushima H, Lima GPP, de Almeida LFR, Barreto P, de Godoy MI (2022) The double knockdown of the mitochondrial uncoupling protein isoforms reveals partial redundant roles during *Arabidopsis thaliana* vegetative and reproductive development. Plant Sci 322:111365. 10.1016/j.plantsci.2022.111365 35779675 10.1016/j.plantsci.2022.111365

[CR37] Long SP, Marshall-Colon A, Zhu XG (2015) Meeting the global food demand of the future by engineering crop photosynthesis and yield potential. Cell 161:56–66. 10.1016/j.cell.2015.03.019 25815985 10.1016/j.cell.2015.03.019

[CR38] Maechler M, Rousseeuw P, Struyf A, Hubert M, Hornik K (2023)Cluster: clusteranalysis basics and extensions. R package version 2.1.6. URL: https://CRAN.R-project.org/package=cluster

[CR39] Masclaux DC, Daniel-Vedele F, Dechorgnat J, Chardon F, Gaufichon L, Suzuki A (2010) Nitrogen uptake, assimilation and remobilization in plants: challenges for sustainable and productive agriculture. Ann Bot 105:1141–1157. 10.1093/aob/mcq028 20299346 10.1093/aob/mcq028 PMC2887065

[CR40] Mathan J, Singh A, Jathar V, Ranjan A (2021a) High photosynthesis rate in the selected wild rice is driven by leaf anatomy mediating high Rubisco activity and electron transport rate. J Exp Bot 72:7119–7135. 10.1093/jxb/erab313 34185840 10.1093/jxb/erab313

[CR41] Mathan J, Singh A, Ranjan A (2021b) Sucrose transport and metabo lism control carbon partitioning between stem and grain in rice. J Exp Bot 72:4355–4372. 10.1093/jxb/erab066 33587747 10.1093/jxb/erab066

[CR42] McAusland L, Atkinson JA, Lawson T, Murchie EH (2019) High throughput procedure utilising chlorophyll fluorescence imaging to phenotype dynamic photosynthesis and photoprotection in leaves under controlled gaseous conditions. Plant Methods 15:e109. 10.1186/s13007-019-0485-x 10.1186/s13007-019-0485-x PMC6749646 31548849

[CR43] Meloni M, Gurrieri L, Fermani S, Velie L, Sparla F, Crozet P, Henri J, Zaffagnini M (2023) Ribulose-1,5-bisphosphate regeneration in the Calvin–Benson–Bassham cycle: focus on the last three enzymatic steps that allow the formation of Rubisco substrate. Front Plant Sci 14:1130430. 10.3389/fpls.2023.1130430 36875598 10.3389/fpls.2023.1130430 PMC9978339

[CR44] Mohidem NA, Hashim N, Shamsudin R, Che Man H (2022) Rice for food security: revisiting its production, diversity, rice milling process and nutrient content. Agriculture 12:741. 10.3390/agriculture12060741

[CR45] Neuhäuser B, Dynowski M, Mayer M, Ludewig U (2007) Regulation of NH4^+^ transport by essential cross talk between AMT monomers through the carboxyl tails. Plant Physiol 143:1651–1659. 10.1104/pp.106.094243 17337531 10.1104/pp.106.094243 PMC1851830

[CR46] Padmavathi G, Bangale U, Rao KN, Balakrishnan D, Arun MN, Singh RK, Sundaram RM (2024) Progress and prospects in harnessing wild relatives for genetic enhancement of salt tolerance in rice. Front Plant Sci 14:1253726. 10.3389/fpls.2023.1253726 38371332 10.3389/fpls.2023.1253726 PMC10870985

[CR47] Phillips AL, Scafaro AP, Atwell BJ (2022) Photosynthetic traits of Australian wild rice (*Oryza australiensis*) confer tolerance to extreme daytime temperatures. Plant Mol Biol 11:347–363. 10.1007/s11103-021-01210-3 10.1007/s11103-021-01210-3 PMC9646608 34997897

[CR48] Popat R, Patel H, Popat P (2024) Agri analyze (Online tool). www.agrianalyze.com

[CR49] Rangan P, Pradheep K, Archak S, Smykal P, Henry R (2023) Editorial: genomics and phenomics of crop wild relatives (CWRs) for crop improvement. Front Plant Sci 14:1221601. 10.3389/fpls.2023.1221601 37332694 10.3389/fpls.2023.1221601 PMC10272818

[CR50] Rangan P, Wankhede DP, Rajkumar S, Chinnusamy V, Pathania P, Bartwal A, Malik SK, Baig MJ, Rai AK, Singh K (2025) Rice transcriptomics reveal the genetic determinants of an *in planta* photorespiratory bypass: a novel way to increase biomass in C_3_ plants. Plant Mol Biol Rep 43:129–138. 10.1007/s11105-024-01469-y

[CR51] Rong L, An J, Chen X, Wang C, Wu J, Wang P, Zheng Y, Wang X, Chai X, Li W, Hu Z, Lu D, Chen GE, Ouyang M, Grimm B, Zhang L, Xu X (2025) LTD coordinates chlorophyll biosynthesis and light-harvesting chlorophyll a/b-binding protein transport. Plant Cell 37:koaf068. 10.1093/plcell/koaf068 40138376 10.1093/plcell/koaf068 PMC11979457

[CR52] Rosado-Souza L, Yokoyama R, Sonnewald U, Fernie AR (2023) Understanding source sink interactions: progress in model plants and translational research to crops. Mol Plant 16:96–121. 10.1016/j.molp.2022.11.015 36447435 10.1016/j.molp.2022.11.015

[CR53] Scafaro AP, Galle A, Van Rie J, Carmo-Silva E, Salvucci ME, Atwell BJ (2016) Heat tolerance in a wild *Oryza* species is attributed to maintenance of Rubisco activation by a thermally stable Rubisco activase ortholog. New Phytol 211:899–911. 10.1111/nph.13963 27145723 10.1111/nph.13963

[CR54] Shahbandeh M (2024) Rice consumption worldwide in 2023/2024, by country (in 1000 metric tons)*. https://www.statista.com/statistics/255971/top-countries-based-on-rice-consumption-2012-2013

[CR55] Sharkey TD (1985) Photosynthesis in intact leaves of C_3_ plants: physics, physiology and rate limitations. Bot Rev 51:53–105. 10.1007/BF02861058

[CR56] Sharkey TD (2016) What gas exchange data can tell us about photosynthesis. Plant Cell Environ 39:1161–1163. 10.1111/pce.12641 26390237 10.1111/pce.12641

[CR57] Shevela D, Bjorn LO, Govinjee G (2019) Photosynthesis: solar energy for life. World Scientific Publishing Co., Singapore. 10.1142/10522

[CR58] Singh A, Mathan J, Dwivedi A, Rani R, Ranjan A (2025) Integration of metabolite and transcriptome profiles of cultivated and wild rice to unveil gene regulatory networks and key genes determining rice source and sink strength. Funct Integr Genom 25:97. 10.1007/s10142-025-01606-0 10.1007/s10142-025-01606-0 40310586

[CR59] Sommer SG, Han E, Li X, Rosenqvist E, Liu F (2023) The chlorophyll fluorescence parameter Fv/Fm correlates with loss of grain yield after severe drought in three wheat genotypes grown at two CO_2_ concentrations. Plants 12:436. 10.3390/plants12030436 36771521 10.3390/plants12030436 PMC9920701

[CR60] Song Y, Yang C, Gao S, Zhang W, Li L, Kuai B (2014) Age-triggered and dark-induced leaf senescence require the bHLH transcription factors PIF3, 4, and 5. Mol Plant 7:1776–1787. 10.1093/mp/ssu109 25296857 10.1093/mp/ssu109 PMC4261840

[CR61] South PF, Cavanagh AP, Lopez-Calcagno PE, Raines CA, Ort DR (2018) Optimizing photorespiration for improved crop productivity. J Integr Plant Biol 60:1217–1230. 10.1111/jipb.12709 30126060 10.1111/jipb.12709

[CR62] Stein JC, Yu Y, Copetti D, Zwickl DJ, Zhang L, Zhang C et al (2018) Genomes of 13 domesticated and wild rice relatives highlight genetic conservation, turnover and innovation across the genus *Oryza*. Nat Genet 50:285–296. 10.1038/s41588-018-0040-0 29358651 10.1038/s41588-018-0040-0

[CR63] Sujatha B (2015) Photosynthesis. In: Bahadur B, Venkat Rajam M, Sahijram L, Krishnamurthy K (eds) Plant BiolBiotech. Springer, New Delhi. 10.1007/978-81-322-2286-6_22

[CR64] Tanaka M, Keira M, Yoon DK et al (2022) Photosynthetic enhancement, lifespan extension, and leaf area enlargement in flag leaves increased the yield of transgenic rice plants overproducing Rubisco under sufficient fertilization. Rice 15:10. 10.1186/s12284-022-00557-5 35138458 10.1186/s12284-022-00557-5 PMC8828814

[CR65] Tsai YC, Chen KC, Cheng TS et al (2019) Chlorophyll fluorescence analysis in diverse rice varieties reveals the positive correlation between the seedlings salt tolerance and photosynthetic efficiency. BMC Plant Biol 19:403. 10.1186/s12870-019-1983-8 31519149 10.1186/s12870-019-1983-8 PMC6743182

[CR66] Uflewski M, Rindfleisch T, Korkmaz K et al (2024) The thylakoid proton antiporter KEA3 regulates photosynthesis in response to the chloroplast energy status. Nat Commun 15:2792. 10.1038/s41467-024-47151-5 38555362 10.1038/s41467-024-47151-5 PMC10981695

[CR67] van Bezouw RFHM, Keurentjes JJB, Harbinson J, Aarts MGM (2019) Converging phenomics and genomics to study natural variation in plant photosynthetic efficiency. Plant J 97:112–133. 10.1111/tpj.14190 30548574 10.1111/tpj.14190 PMC6850172

[CR68] Van Dijk M, Morley T, Rau ML, Saghai Y (2021) A meta-analysis of projected global food demand and population at risk of hunger for the period 2010–2050. Nat Food 2:494–501. 10.1038/s43016-021-00322-9 37117684 10.1038/s43016-021-00322-9

[CR69] Walkowiak S, Gao L, Monat C, Haberer G, Kassa MT et al (2020) Multiple wheat genomes reveal global variation in modern breeding. Nature 588:277–283. 10.1038/s41586-020-2961-x 33239791 10.1038/s41586-020-2961-x PMC7759465

[CR70] Xiong D (2024) Perspectives of improving rice photosynthesis for higher grain yield. Crop Environ 3:123–137. 10.1016/j.crope.2024.04.001

[CR71] Yiwei F, Jiayelu W, Mingming W, Shenghai Y, Rongrong Z, Jing Y, Guofu Z, Faming Y, Yanting L, Xiaoming Z (2024) Progress on molecular mechanism of heat tolerance in rice. Rice Sci 31:673–687. 10.1016/j.rsci.2024.07.001

[CR72] Yuan J, Zhong S, Long Y, Guo J, Yu Y, Liu J (2022) Shikimate kinase plays important roles in anthocyanin synthesis in Petunia. Int J Mol Sci 23:15964. 10.3390/ijms232415964 36555606 10.3390/ijms232415964 PMC9786173

[CR73] Zhang H, Chen J, Liu X, Wang R, Zhang H, Yang Y (2025) Enhancing rice (*Oryza sativa* L.) yield and quality by improving photosynthesis with foliar application of zinc oxide nanoparticles. Environ Sci Nano 12:2331–2342. 10.1039/D4EN01209G

