# Supplementary material for: Exploring wild rices for photosynthetic efficiency improvement in rice
Source: Physiol Mol Biol Plants. 2026 Jun 13;32(6):1261–76. doi: 10.1007/s12298-026-01761-z (PMC13291300; doi:10.1007/s12298-026-01761-z)
Supplement: Supplementary file 1 — Supplementary Material 1 [file 12298_2026_1761_MOESM1_ESM.docx]

**Table S1** Chromosome number and genomic composition of *Oryza* species.

| **S. No** | | | **Species** | **Accession no.** | **2 n** | **Genome** |
| --- | --- | --- | --- | --- | --- | --- |
|  | | ***O*. *sativa complex*** | | | | |
| 1. | *O*. *sativa* L.(subspecies, *indica*, *japonica*, and *javanica*) | | |  | 24 | AA |
| 2. | *O*. *glaberrima* Steud | | |  | 24 | A ^g^ A ^g^ |
| 3. | *O*. *nivara* Sharma et Shastry | | | IRGC 182018 | 24 | AA |
| 4. | *O*. *ruﬁpogon* Griff | | | IRGC -103404 | 24 | AA |
| 5. | *O. spontanea* | | | IC 0616007 | 24 | AA |
| 6. | *O. barthii* A. Chev et Roehr. *O.* | | | EC 946900 | 24 | A ^g^ A ^g^ |
| 7. | *O. longistaminata* A. Chev et Roehr | | | EC 946895 | 24 | A ^l^ A ^l^ |
| 8. | *O. meridionalis* Ng | | |  | 24 | *A* ^m^ *A* ^m^ |
| 9. | *O*. *glumaepatula* Steud | | |  | 24 | A^gp^A^gp^ |
|  | | ***O. ofﬁcinalis* complex** | | | | |
| 10. | *O*. *punctata* Kotschy ex Steud*.* | | | EC 946906 | 24 ,48 | BB, BBCC |
| 11. | *O*. *minuta* J. S. Presl. ex C.B. Presl. | | | EC 946907 | 48 | BBCC |
| 12. | *O*. *ofﬁcinalis* Wall ex Watt | | |  | 24 | CC |
| 13. | *O*. *rhizomatis* Vaughan | | |  | 24 | CC |
| 14. | *O*. *eichingeri A.* Peter | | | IRGC-101424 | 24 | CC |
| 15. | *O*. *latifolia* Desv | | | IRGC-103787 | 48 | CCDD |
| 16. | *O*. *alta* Swallen | | | EC 946910 | 48 | CCDD |
| 17. | *O*. *grandiglumis* (Doell) Prod. | | | EC 946904 | 48 | CCDD |
| 18. | *O*. *australiensis* Domin. | | | EC 946897 | 24 | EE |
|  | |  | | | | |
| 19. | *O*. *granulata* Nees et Arn. ex Watt | | | EC 946908 | 24 | GG |
| 20. | *O*. *meyeriana* (Zoll. et (Mor. ex Steud.) Baill.) | | | IRGC-104990 | 24 | GG |
|  | | ***O*. *ridleyi* complex** | | | | |
| 21. | *O*. *longiglumis* Jansen | | |  | 48 | HHJJ |
| 22. | *O*. *ridleyi* Hook. F. | | | EC 946899 | 48 | HHJJ |
|  | | **Unclassiﬁed** | | | | |
| 23. | *O*. *brachyantha A .*Chev et Roehr | | |  | 24 | FF |
| 24. | *O*. *coarctata* Tateoka | | |  | 48 | KKLL |

**Table S2 (A) Anova for photosynthesis traits**

| **Sources of variations** | **df** | **SS** | **MS** | ***F*** | **P value** | **Result** |
| --- | --- | --- | --- | --- | --- | --- |
| **Photosynthesis** | | | | | | |
| **Replication** | 2 | 5.304 | 2.65 | 1.618 | 0.2083 | NS |
| **Species** | 25 | 2672.9 | 106.9 | 65.2** | 1.1x10^-16^ | ** Sig. at 1% level |
| **Error** | 50 | 81.9 | 1.63 |  |  |  |
| **Stomatal conductance** | | | | | | |
| **Replication** | 2 | 0.00081 | 0.0041 | 3.6516* | 0.0331 | * Sig. at 5% level |
| **Species** | 25 | 0.7119 | 0.0285 | 25.62** | 1.1x10^-16^ | ** Sig. at 1% level |
| **Error** | 50 | 0.0556 | 0.0011 |  |  |  |
| **Intercellular CO_2_** | | | | | | |
| **Replication** | 2 | 1248.5 | 624.14 | 4.34* | 0.0181 | * Sig. at 5% level |
| **Species** | 25 | 72275.45 | 2891.02 | 20.14** | 1.1x10^-16^ | ** Sig. at 1% level |
| **Error** | 50 | 7176.36 | 143.53 |  |  |  |
| **Transpiration** | | | | | | |
| **Replication** | 2 | 4.61 | 2.30 | 6.91** | 0.0022 | ** Sig. at 1% level |
| **Species** | 25 | 194.40 | 7.78 | 23.32** | 1.1x10^-16^ | ** Sig. at 1% level |
| **Error** | 50 | 16.67 | 0.33 |  |  |  |
| **Water use efficiency** | | | | | | |
| **Replication** | 2 | 1.413 | 0.7065 | 1.8741 | 0.1641 | NS |
| **Species** | 25 | 64.776 | 2.591 | 6.8729 | 4.3x10-^09^ | ** Sig. at 1% level |
| **Error** | 50 | 18.849 | 0.3770 |  |  |  |
| **Carboxylation efficiency** | | | | | | |
| **Replication** | 2 | 0.00013 | 0.00007 | 1.69 | 0.1974 | NS |
| **Species** | 25 | 0.04063 | 0.00163 | 41.23** | 1.1x10-^16^ | ** Sig. at 1% level |
| **Error** | 50 | 0.00197 | 0.00004 |  |  |  |
| **Ci/Ca** | | | | | | |
| **Replication** | 2 | 0.0032 | 0.0016 | 1.78 | 0.1798 | NS |
| **Species** | 25 | 0.4079 | 0.0163 | 18.03** | 1.1x10^-16^ | ** Sig. at 1% level |
| **Error** | 50 | 0.0453 | 0.00091 |  |  |  |

**Table S2 (B) Statistics for photosynthesis traits**

| **Statistic** | **PN** | **gs** | **Ci** | **E** | **WUE** | **CE** | **Ci/Ca** |
| --- | --- | --- | --- | --- | --- | --- | --- |
| **Grand Total** | 1170.249 | 15.2430 | 20626.4830 | 340.0200 | 277.85 | 4.5050 | 50.337 |
| **Grand Mean** | 15.0032 | 0.1954 | 264.4421 | 4.3592 | 3.5622 | 0.0578 | 0.6453 |
| **Maximum** | 26.316 | 0.644 | 351.99 | 9.4 | 8.174 | 0.1 | 0.833 |
| **Minimum** | 5.184 | 0.057 | 181.083 | 1.149 | 1.547 | 0.016 | 0.438 |
| **Correction Factor** | 17557.4708 | 2.9788 | 5454510.2686 | 1482.2256 | 989.7516 | 0.2602 | 32.4848 |
| **SEm** | 0.7390 | 0.0192 | 6.9168 | 0.3334 | 0.3545 | 0.0037 | 0.0174 |
| **SEd** | 1.0451 | 0.0272 | 9.7818 | 0.4715 | 0.5013 | 0.0052 | 0.0246 |
| **CD at 5%** | 2.0992 | 0.0547 | 19.6473 | 0.947 | 1.007 | 0.0104 | 0.0493 |
| **CD at 1%** | 2.7986 | 0.0729 | 26.1936 | 1.2625 | 1.3425 | 0.0139 | 0.0658 |
| **C.V** | 8.5317 | 17.0592 | 4.5304 | 13.2461 | 17.2367 | 10.9842 | 4.661 |
| **Env. Variance** | 1.6385 | 0.0011 | 143.5255 | 0.3334 | 0.3770 | 4.0 x 10^-5^ | 0.0009 |
| **Genotypic Variance** | 35.0934 | 0.0091 | 915.8262 | 2.4809 | 0.7380 | 0.0005 | 0.0051 |
| **Phenotypic Variance** | 36.7319 | 0.0102 | 1059.3517 | 2.8143 | 1.1150 | 0.0006 | 0.0060 |
| **Heritability** | 0.9554 | 0.8914 | 0.8645 | 0.8815 | 0.6619 | 0.9295 | 0.8502 |
| **Genotypic Coefficient of Variance** | 39.4847 | 48.8712 | 11.4440 | 36.1320 | 24.1168 | 39.8895 | 11.1032 |
| **Phenotypic Coefficient of Variance** | 40.3960 | 51.7630 | 12.3081 | 38.4835 | 29.6433 | 41.3743 | 12.0419 |
| **Env. Coefficient of Variance** | 8.5317 | 17.0592 | 4.5304 | 13.2461 | 17.2367 | 10.9842 | 4.6610 |
| **Genetic Advance** | 11.9281 | 0.1858 | 57.9642 | 3.0464 | 1.4398 | 0.0458 | 0.1361 |
| **GA as % of Mean** | 79.5038 | 95.0503 | 21.9194 | 69.8837 | 40.4185 | 79.2237 | 21.0898 |

**Table S3 (A) Anova for chlorophyll fluorescence traits**

| **Sources of variations** | **df** | **SS** | **MS** | ***F*** | **P value** | **Result** |
| --- | --- | --- | --- | --- | --- | --- |
| **Fv′/Fm′** | | | | | | |
| **Replication** | 2 | 0.0019 | 0.00096 | 1.53 | 0.2277 | NS |
| **Species** | 25 | 0.351 | 0.01404 | 22.39** | 1.1x10^-16^ | ** Sig. at 1% level |
| **Error** | 50 | 0.0314 | 0.00063 |  |  |  |
| **Photochemical quenching** | | | | | | |
| **Replication** | 2 | 0.0184 | 0.0092 | 5.24** | 0.0084 | ** Sig. at 1% level |
| **Species** | 25 | 0.0844 | 0.0337 | 19.22** | 1.1x10^-16^ | ** Sig. at 1% level |
| **Error** | 50 | 0.088 | 0.0017 |  |  |  |
| **Non-photochemical quenching** | | | | | | |
| **Replication** | 2 | 0.1831 | 0.0916 | 5.551** | 0.0069 | ** Sig. at 1% level |
| **Species** | 25 | 3.473 | 0.1389 | 8.36** | 1.4x10^-10^ | ** Sig. at 1% level |
| **Error** | 50 | 0.831 | 0.0166 |  |  |  |
| **ΦPSII** | | | | | | |
| **Replication** | 2 | 0.0010 | 0.0005 | 3.1556 | 0.0512 | NS |
| **Species** | 25 | 0.1399 | 0.0056 | 35.3358** | 1.1x10^-16^ | ** Sig. at 1% level |
| **Error** | 50 | 0.0079 | 0.0002 |  |  |  |
| **Electron transport rate** | | | | | | |
| **Replication** | 2 | 232.16 | 116.08 | 3.01 | 0.0582 | NS |
| **Species** | 25 | 37032.7 | 1481.31 | 38.43** | 1.1x10^-16^ | ** Sig. at 1% level |
| **Error** | 50 | 1927.42 | 38.548 |  |  |  |

**Table S3 (B) Statistics for chlorophyll fluorescence traits.**

| **Statistic** | **Fv′/Fm′** | **qP** | **qN** | **ΦPSII** | **ETR** |
| --- | --- | --- | --- | --- | --- |
| **Grand Total** | 36.13 | 24.666 | 142.681 | 10.9510 | 5758.485 |
| **Grand Mean** | 0.4632 | 0.3162 | 1.8292 | 0.1404 | 73.827 |
| **Maximum** | 0.611 | 0.554 | 2.573 | 0.222 | 116.47 |
| **Minimum** | 0.317 | 0.058 | 1.411 | 0.052 | 27.273 |
| **Correction Factor** | 16.7356 | 7.8001 | 260.9983 | 1.5375 | 425130.1217 |
| **SEm** | 0.0145 | 0.0242 | 0.0744 | 0.0073 | 3.5846 |
| **SEd** | 0.0205 | 0.0342 | 0.1053 | 0.0103 | 5.0694 |
| **CD at 5%** | 0.0411 | 0.0687 | 0.2114 | 0.0206 | 10.1822 |
| **CD at 1%** | 0.0548 | 0.0916 | 0.2818 | 0.0275 | 13.5748 |
| **C.V** | 5.4093 | 13.2492 | 7.0472 | 8.9645 | 8.4098 |
| **Env. Variance** | 0.0006 | 0.0018 | 0.0166 | 0.0002 | 38.5481 |
| **Genotypic Variance** | 0.0045 | 0.0107 | 0.0408 | 0.0018 | 480.9200 |
| **Phenotypic Variance** | 0.0051 | 0.0124 | 0.0574 | 0.0020 | 519.4681 |
| **Heritability** | 0.8767 | 0.8587 | 0.7104 | 0.9196 | 0.9258 |
| **Genotypic Coefficient of Variance** | 14.4220 | 32.6581 | 11.0371 | 30.3277 | 29.7045 |
| **Phenotypic Coefficient of Variance** | 15.4030 | 35.2433 | 13.0950 | 31.6248 | 30.8721 |
| **Env. Coefficient of Variance** | 5.4093 | 13.2492 | 7.0472 | 8.9645 | 8.4098 |
| **Genetic Advance** | 0.1288 | 0.1971 | 0.3505 | 0.0841 | 43.4671 |
| **GA as % of Mean** | 27.8169 | 62.3406 | 19.1632 | 59.9124 | 58.8772 |

**Table S4 MANOVA results photosynthesis and associated traits**

| **Statistic** | **Df** | **Value** | **Approx. F** | **Numerator df** | **Denominator df** | **Pr(>F)** |
| --- | --- | --- | --- | --- | --- | --- |
| **Pillai’s Trace**  **Wilks**  **Hotelling-Lawley Trace**  **Roy’s Greatest Root** | 25  25  25  25 | 8.5036  5.6598x10^-10^  150.44  81.479 | 5.0587  9.7898  19.641  169.48 | 300  300  300  25 | 624  481  470  52 | < 2.2x 10^-16^ *******  < 2.2x 10^-16^ *******  < 2.2x 10^-16^ *******  < 2.2x 10^-16^ ******* |
|  |  |  |  |  |  |  |

* Sig. at 5% level; ** Sig. at 1% level;*** Sig. at 0.1% level

**Table S5 PERMANOVA results for photosynthesis and associated traits (based on 999 permutations)**

| **Sources of variations** | **df** | **SS** | **R^2^** | ***F*** | **P value** |  |
| --- | --- | --- | --- | --- | --- | --- |
| **Photosynthesis** | | | | | | |
| **Model** | 1 | 115.46 | 0.38487 | 15.016 | 0.001 *** |  |
| **Residual** | 24 | 184.54 | 0.61513 |  |  |  |
| **Total** | 25 | 300.00 | 1.00000 |  |  |  |
| **Stomatal conductance** | | | | | | |
| **Model** | 1 | 91.911 | 0.30637 | 10.601 | 0.001 *** |  |
| **Residual** | 24 | 208.089 | 0.69393 |  |  |  |
| **Total** | 25 | 300.000 | 1.00000 |  |  |  |
| **Intercellular CO_2_** | | | | | | |
| **Model** | 1 | 43.782 | 0.14594 | 4.101 | 0.011 * |  |
| **Residual** | 24 | 256.218 | 0.85406 |  |  |  |
| **Total** | 25 | 300.000 | 1.00000 |  |  |  |
| **Transpiration** | | | | | | |
| **Model** | 1 | 98.353 | 0.32784 | 11.706 | 0.001 *** |  |
| **Residual** | 24 | 201.647 | 0.67216 |  |  |  |
| **Total** | 25 | 300.000 | 1.00000 |  |  |  |
| **Water use efficiency** | | | | | | |
| **Model** | 1 | 98.313 | 0.32771 | 11.699 | 0.001 *** |  |
| **Residual** | 24 | 201.687 | 0.67229 |  |  |  |
| **Total** | 25 | 300.000 | 1.00000 |  |  |  |
| **Carboxylation efficiency** | | | | | | |
| **Model** | 1 | 31.611 | 0.10537 | 2.8268 | 0.047 * |  |
| **Residual** | 24 | 268.389 | 0.89463 |  |  |  |
| **Total** | 25 | 300.000 | 1.00000 |  |  |  |
| **Ci/Ca** | | | | | | |
| **Model** | 1 | 106.17 | 0.3539 | 13.146 | 0.001 *** |  |
| **Residual** | 24 | 193.83 | 0.6461 |  |  |  |
| **Total** | 25 | 300.00 | 1.0000 |  |  |  |
|  |  |  | **Fv′/Fm′** |  |  |  |
| **Model** | 1 | 106.17 | 0.3539 | 13.146 | 0.001 *** |  |
| **Residual** | 24 | 193.83 | 0.6461 |  |  |  |
| **Total** | 25 | 300.00 | 1.0000 |  |  |  |
|  |  |  | **Photochemical quenching** |  |  |  |
| **Model** | 1 | 115.46 | 0.38487 | 15.016 | 0.001 *** |  |
| **Residual** | 24 | 184.54 | 0.61513 |  |  |  |
| **Total** | 25 | 300.00 | 1.00000 |  |  |  |
|  |  |  | **Non-photochemical quenching** |  |  |  |
| **Model** | 1 | 35.822 | 0.11941 | 3.2543 | 0.035 * |  |
| **Residual** | 24 | 264.178 | 0.88059 |  |  |  |
| **Total** | 25 | 300.000 | 1.00000 |  |  |  |
|  |  |  | **ΦPSII** |  |  |  |
| **Model** | 1 | 33.411 | 0.11137 | 3.0078 | 0.02 * |  |
| **Residual** | 24 | 266.589 | 0.88863 |  |  |  |
| **Total** | 25 | 300.000 | 1.00000 |  |  |  |
|  |  |  | **Electron transport rate** |  |  |  |
| **Model** | 1 | 55.293 | 0.18431 | 5.423 | 0.004 ** |  |
| **Residual** | 24 | 244.707 | 0.81569 |  |  |  |
| **Total** | 25 | 300.000 | 1.00000 |  |  |  |
|  |  |  |  |  |  |  |
|  |  |  |  |  |  |  |
|  |  |  |  |  |  |  |
|  |  |  |  |  |  |  |

* Sig. at 5% level; ** Sig. at 1% level;*** Sig. at 0.1% level
